# Vitamin C May Improve Left Ventricular Ejection Fraction: A Meta-Analysis

**DOI:** 10.3389/fcvm.2022.789729

**Published:** 2022-02-25

**Authors:** Harri Hemilä, Elizabeth Chalker, Angelique M. E. de Man

**Affiliations:** ^1^Department of Public Health, University of Helsinki, Helsinki, Finland; ^2^Biological Data Science Institute, Australian National University, Canberra, ACT, Australia; ^3^Department of Intensive Care Medicine, Amsterdam University Medical Centers, Amsterdam, Netherlands

**Keywords:** antioxidant, coronary artery bypass graft surgery (CABG), heart failure, left ventricular function, oxidative stress, percutaneous coronary intervention (PCI), randomized trials, systematic review

## Abstract

**Background:**

Vitamin C deprivation can lead to fatigue, dyspnea, oedema and chest pain, which are also symptoms of heart failure (HF). In animal studies vitamin C has improved contractility and mechanical efficiency of the heart. Compared with healthy people, patients with HF have lower vitamin C levels, which are not explained by differences in dietary intake levels, and more severe HF seems to be associated with lower plasma vitamin C levels. This meta-analysis looks at the effect of vitamin C on left ventricular ejection fraction (LVEF).

**Methods:**

We searched for trials reporting the effects of vitamin C on LVEF. We assessed the quality of the trials, and pooled selected trials using the inverse variance, fixed effect options. We used meta-regression to examine the association between the effect of vitamin C on LVEF level and the baseline LVEF level.

**Results:**

We identified 15 trials, three of which were excluded from our meta-analysis. In six cardiac trials with 246 patients, vitamin C increased LVEF on average by 12.0% (95% CI 8.1–15.9%; *P* < 0.001). In six non-cardiac trials including 177 participants, vitamin C increased LVEF on average by 5.3% (95% CI 2.0–8.5%; *P* = 0.001). In meta-regression analysis we found that the effect of vitamin C was larger in trials with the lowest baseline LVEF levels with *P* = 0.001 for the test of slope. The meta-regression line crossed the null effect level at a baseline LVEF level close to 70%, with progressively greater benefit from vitamin C with lower LVEF levels. Some of the included trials had methodological limitations. In a sensitivity analysis including only the four most methodologically sound cardiac trials, the effect of vitamin C was not substantially changed.

**Conclusions:**

In this meta-analysis, vitamin C increased LVEF in both cardiac and non-cardiac patients, with a strong negative association between the size of the vitamin C effect and the baseline LVEF. Further research on vitamin C and HF should be carried out, particularly in patients who have low LVEF together with low vitamin C intake or low plasma levels. Different dosages and different routes of administration should be compared.

## Introduction

In the 18th century, James Lind described extreme intolerance for exercise as a characteristic of scorbutic patients (1). In addition, other signs compatible with heart failure (HF) such as shortness of breath, lethargy, and swelling of legs were described as symptoms of vitamin C deficiency in the major monographs on scurvy (1, 2). Autopsies of patients who died of scurvy revealed cardiac hypertrophy and congestion of the lungs (2). Experimental vitamin C deprivation in healthy volunteers led to fatigue, dyspnea, oedema, chest pain and reduced autonomic reflexes (3–8).

Case reports of patients with severe vitamin C deficiency have reported fatigue, dyspnea, cardiac enlargement, oedema and orthostatic hypotension, which often disappeared quite rapidly after vitamin C administration (9–15). A few animal models found that vitamin C can improve contractility and mechanical efficiency of the heart, including effects on left ventricular ejection fraction (LVEF) (16–21). Given the overlap of the symptoms of vitamin C deficiency with the symptoms of HF, and the findings from the animal studies, it seems appropriate to investigate whether administration of vitamin C may be beneficial for some HF patients.

Vitamin C exerts a multitude of biochemical effects influencing several cardiovascular processes relevant for HF. It participates in the synthesis of norepinephrine, carnitine, nitric oxide, and in the terminal amidation of dozens of neuropeptides such as vasopressin (22–25). Vitamin C hydroxylates specific proline residues in hypoxia-inducible factor-I, which regulates hundreds of genes, and it also participates in the demethylation of DNA and histones and thereby influences the epigenome (23–26).

In randomized trials, vitamin C has affected the cardiovascular system in various ways. Meta-analyses have indicated that in some contexts vitamin C may reduce blood pressure and risk of atrial fibrillation (27, 28). It has also improved endothelial functions and baroreflex sensitivity (29–36). In patients with ischemia/reperfusion injury, vitamin C may reduce reperfusion damage with amelioration of myocardial stunning (37). Through these types of mechanisms, vitamin C may impact HF.

The goal of this meta-analysis was to analyze the findings of trials that have reported on the effect of vitamin C on LVEF as a measure of the mechanical function of the heart.

## Results

### Description of the Included Trials

**Figure 1:**
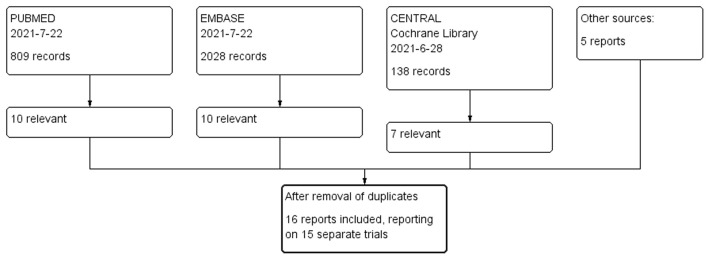
Flow diagram of the searches, which identified 16 publications reporting 15 trials on the effect of vitamin C on LVEF (38–53). The search terms are listed in Supplementary File 1.

We identified 16 publications that reported 15 separate trials (Figure 1, Table 1, Supplementary Table S1). Seven of them were parallel group trials and five were cross-over trials (Table 1). Three further trials were before-after trials: LVEF was first measured at baseline and then a second LVEF measurement was carried out following vitamin C administration of 1 month (49) or 6 months (38). The Basili et al. trial was published in two reports (43, 44) and the Fernhall et al. trial in three reports (45–47). Sabri et al. (49) and Scalzo et al. (52) reported two separate trials in one paper. The total number of patients in the 15 trials was 469, with 246 participants in six cardiac trials, and 223 participants in nine non-cardiac trials, six of which were included in the meta-analysis.

**Table 1:** Characteristics of included trials.

**Trial [ref]**	**N (vit C/ Control)**	**Age (y, mean)**	**Country**	**Context**	**LVEF at baseline^a^**	**Vit C route**	**Vit C dose (g/day)**	**Vit C days**
**Cardiac trials**								
Guan et al. (39)	10/11	65	Japan	PCI	50%	iv	6	1
Oktar et al. (40)	12/12	56	Turkey	CABG	61.5%	iv	4	1
Ho (41)	37^b^	69	Taiwan	HF	35%	po	4	28
Basili et al. (43) Pignatelli et al. (44)	28/28	67	Italy	PCI	53%	iv	1	1
Safaei et al. (51)	29/29	57	Iran	CABG	49%	iv	2	1
Emadi et al. (53)	25/25	62	Iran	CABG	56%	iv	10	1
**Non-cardiac trials**								
Jensen et al. (38)	9^c^	52	Denmark	Iron-loaded adults	56%	po	0.2	180
Glavas et al. (42)	8^b^	37	Croatia	Diving	66%	po	1	1
Fernhall et al. (45) Fahs et al. (46) Fernhall et al. (47)	34/35	28	USA	Fire fighters	59%	po	2	1
Gao et al. (48)	8^d^	26	USA	Hyperoxia	59%	iv	3	1
Sabri et al. (49)	19^c^	13	Iran	Healthy	66%	po	0.25	30
Sabri et al. (49)	18^c^	11	Iran	T1D	66%	po	0.25	30
Sabri et al. (50)	20/20	13	Iran	T1D	60%	po	0.25	180
Scalzo et al. (52)	21^b^	45	USA	Exercise; Healthy	66%	iv	7.5	1
Scalzo et al. (52)	31^b^	46	USA	Exercise; T2D	64%	iv	7.5	1

a *Baseline LVEF is the mean of vitamin C and control groups*.

b *Cross-over; random order*.

c *Before-after study*.

d *Cross-over; first was the control test and thereafter the vitamin C test*.

*CABG, coronary artery bypass graft surgery; iv, intravenous; LVEF, left ventricular ejection fraction; PCI, percutaneous coronary intervention; po, per oral; T1D, type 1 diabetes; T2D, type 2 diabetes*.

In the six cardiac trials, the mean age ranged from 56 to 69 years, and the baseline LVEF ranged from 35 to 62%. Five of the cardiac trials investigated the effect of a single intravenous dose of vitamin C before or during cardiac surgery with the dose ranging from 1 to 10 g (39, 40, 43, 51, 53). A trial with HF patients used a cross-over design to investigate the effect of 4-week oral administration of vitamin C at 4 g/day compared with placebo (41).

The nine non-cardiac LVEF trials form a heterogeneous group of studies. The mean age of participants ranged from 11 to 52 years. The baseline LVEF was rather high in all these trials, ranging from 56 to 66%. In children with diabetes, Sabri et al. administered 0.25 g/day of vitamin C orally for 6 months (50). In two cross-over trials published in one trial report, Scalzo et al. administered 7.5 g of vitamin C intravenously before an exercise test for type 2 diabetes (T2D) patients and matched healthy participants (52). In a cross-over trial, Gao et al. studied the effect of 3 g/day intravenous vitamin C on cardiac effects of hyperoxia (48). In one before-after trial, Jensen et al. administered 0.2 g/day of vitamin C orally for 12 months to adults who suffered from transfusion-induced iron overload (38), and in two before-after trials, Sabri et al. gave vitamin C to healthy participants and to type 1 diabetes mellitus (T1D) patients (49).

**Figure 2:**
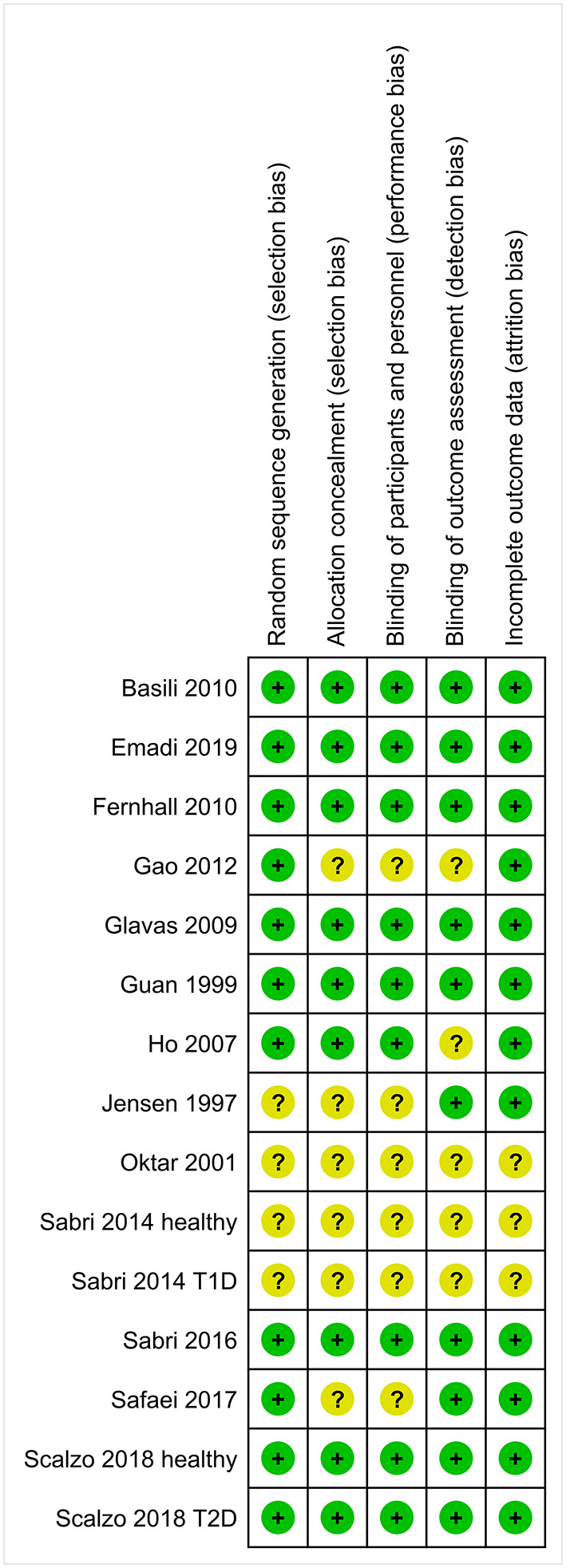
Risk of bias summary of the trials that reported on the effect of vitamin C on LVEF. Review authors' judgments are shown for each risk of bias item for each included trial. A green plus sign (+) indicates that there is no substantial concern for bias in the particular quality item. A question mark (?) indicates that conclusions are unable to be drawn regarding potential bias. The reference numbers to the trials are shown in Table 1. Justifications for the quality assessments are described in Supplementary Table S1.

The risk of bias assessment for the 15 included trials is shown in Figure 2. Eleven trials were randomized. Oktar et al. did not describe the method of allocation in their CABG trial (40). In three self-controlled trials, a baseline LVEF measurement was taken, followed by a period of vitamin C treatment, and then a second LVEF measurement was taken (38, 49). The reported baseline variables for the treatment groups in the parallel-group trials were balanced (Supplementary Table S1). Eight trials used an explicit placebo (41–43, 45, 50, 52, 53), whereas four trials reported “no treatment” in the control groups (39, 40, 48, 51). The trials by Gao et al. (48), Jensen et al. (38), Oktar et al. (40), Safaei et al. (51), and Sabri et al. (49) had the most methodological concerns (Figure 2). The Jensen et al. trial (38) and the both Sabri et al. (49) trials were excluded from our meta-analysis as they did not have an explicit control group or control treatment period.

In 11 trials LVEF was measured by transthoracic echocardiography (40–50, 52, 53). Biplane Simpson's rule was used to calculate LVEF in 3 trials (41, 43, 48), 2-D guided M-mode measurement of the cross-sectional axis of the LV at the papillary muscle tip level in 1 trial (42) and in the other 7 trials this was not specified (40, 45–47, 49, 50, 52, 53). Jensen et al. (38) estimated LVEF by resting multigated acquisition (MUGA) scans, Oktar et al. (40) by both MUGA scan and echocardiography and Guan et al. by left ventriculography (39). Safaei et al. did not describe the method of measurement of LVEF in their CABG trial (51).

### Results of the Included Trials

#### Vitamin C and LVEF

For our quantitative study, we excluded three before-after trials (38, 49), restricting the meta-analysis to seven parallel group trials and five cross-over trials – 12 trials in total.

**Figure 3:**
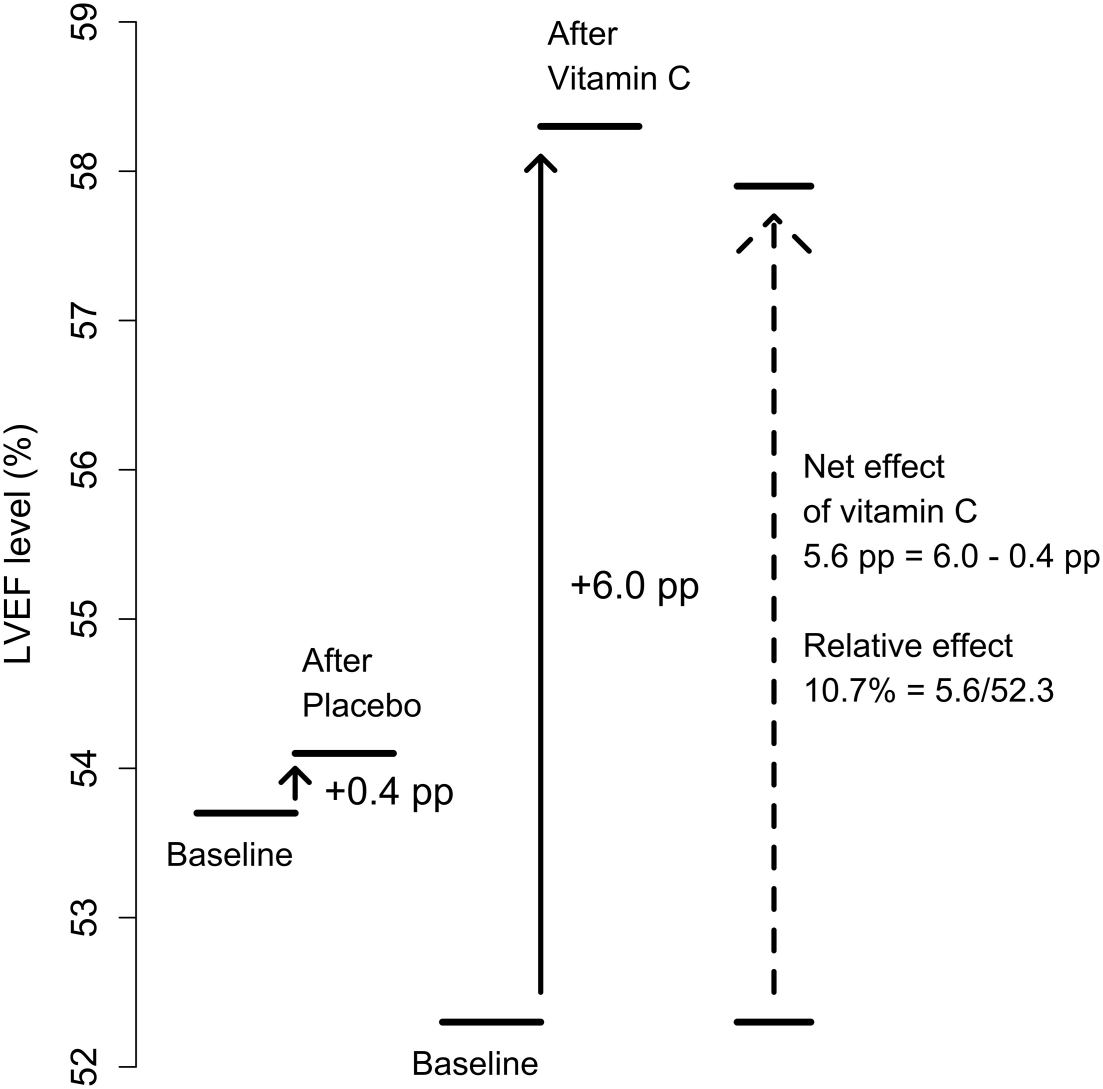
Calculation of the effect of vitamin C on LVEF. The calculation is illustrated with figures from the Basili et al. trial during PCI (percutaneous coronary intervention) (43). In the placebo group, LVEF was increased from a baseline level of 53.7 to 54.1% after the intervention, while in the vitamin C group from 52.3 to 58.3%. These correspond to 0.4 and 6.0 percentage point (pp) increases in LVEF, respectively. Thus, subtracting the placebo group change from the vitamin C group change gives the net effect of vitamin C as 5.6 pp. On the relative scale this corresponds to a 10.7% (= 5.6/52.3) increase in LVEF due to vitamin C from the baseline level.

**Figure 4:**
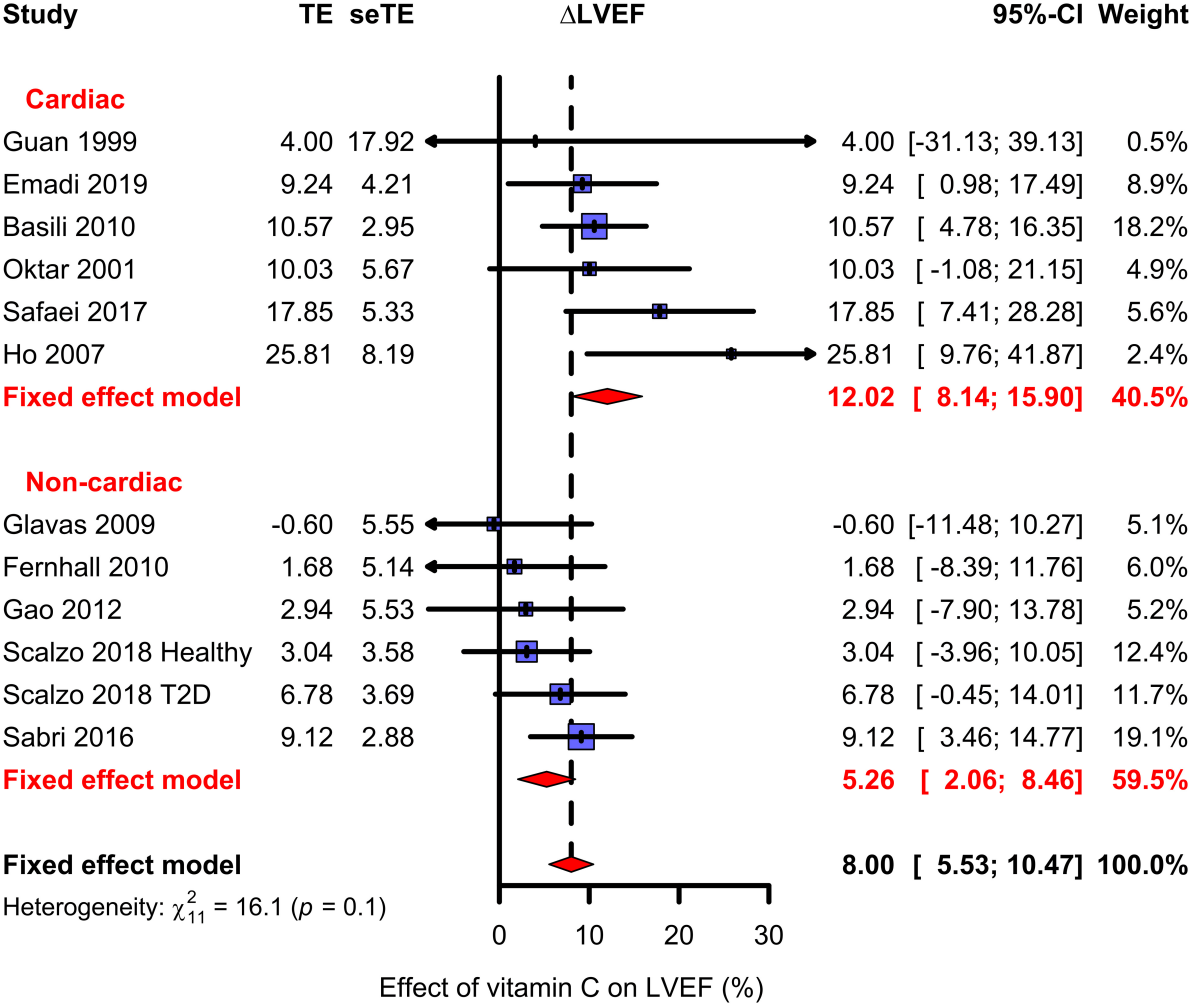
Effect of vitamin C on LVEF. The upper subgroup shows the cardiac trials and the lower subgroup shows the non-cardiac trials. The effect of vitamin C is shown as the percentage difference between the vitamin C and placebo groups from the baseline LVEF; see Figure 3. The horizontal lines indicate the 95% CI for the vitamin C effect and the blue squares in the middle of the horizontal lines indicate the point estimate of the effect in the particular trial. The size of the blue square reflects the weight of the trial in the meta-analysis. The red diamond shape indicates the pooled effect and 95% CI for the two subgroups and for all 12 trials. See Supplementary Files 1, 2 for the description of the trials and the calculations.

We calculated the difference in LVEF changes between the parallel vitamin C and control groups, and between the vitamin C and control periods in the cross-over trials. We used the relative scale which has been shown to be superior in the analysis of continuous outcomes (54–56). See Figure 3 for an illustration of the calculation for the effect of vitamin C on LVEF. A similar approach was previously used in the analysis of changes in FEV_1_ levels in β_2_-agonist trials (54). We analyzed the findings of cardiac and non-cardiac trials separately (Figure 4, Table 1).

In six cardiac trials with 246 patients, vitamin C increased LVEF on average by 12.0% (95% CI 8.1–15.9%; *P* = 10^−9^) (Figure 4). In four trials, the benefit was statistically significant. There was no evidence of heterogeneity between the six cardiac trials (*P* = 0.4). There are methodological concerns with the Oktar and Safaei trials (Figure 2). Nevertheless, if these two trials are excluded in a sensitivity analysis, the estimate of vitamin C effect changes only slightly to 11.3% (95% CI 6.7–15.8%; *P* = 10^−6^). The methodologically sound Basili trial has the greatest weight in the cardiac meta-analysis, with an estimate of effect close to the pooled effect in the cardiac group (Figure 4). Thus, there is no indication that methodologically inferior trials exaggerate the estimate of effect.

In the meta-analysis of six non-cardiac trials including 177 participants, vitamin C increased LVEF on average by 5.3% (95% CI 2.0–8.5%; *P* = 0.0013) (Figure 4). A statistically significant benefit was found in two of the individual trials. There was no evidence of heterogeneity (*P* = 0.5).

In explorative meta-regression analyses, we investigated a few relevant variables over the 12 trials as follows. There was no evidence of a difference in estimates between four trials with oral administration and eight trials with intravenous administration (*P* = 0.8). In meta-regression, the dose of vitamin C was not associated with the size of the effect (*P* = 0.7). For example, Basili (43) administered 1 g and Emadi (53) administered 10 g on one single day, but the reported effects were quite similar (Figure 4). See Supplementary File 1 for the analyses.

**Figure 5:**
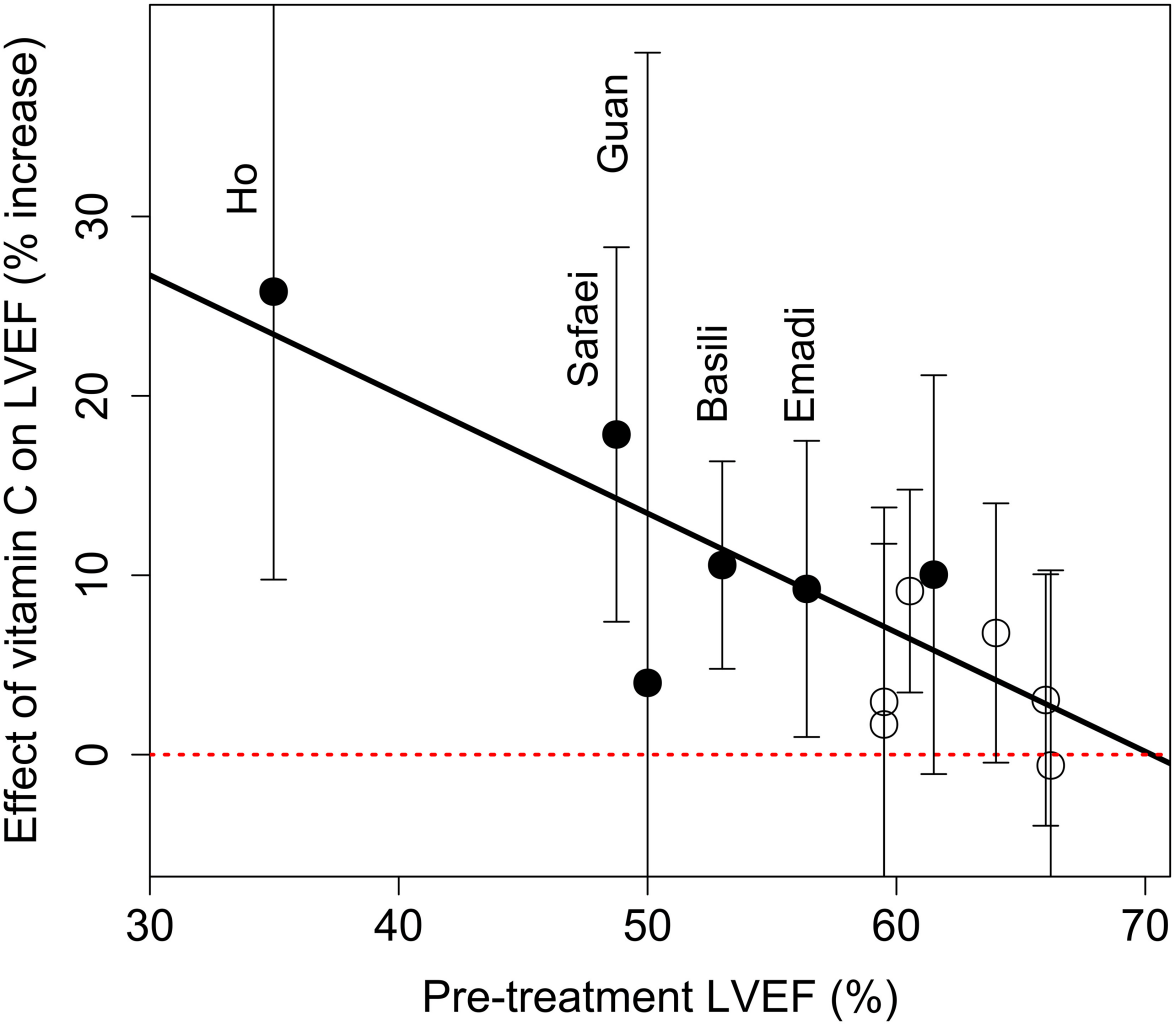
Effect of vitamin C on LVEF by the baseline LVEF. The effect of vitamin C is shown as the percentage differences between the vitamin C and control groups from the baseline LVEF. The vertical lines indicate the 95% CI for the vitamin C effect in each trial. The cardiac trials are indicated by filled circles, and the non-cardiac trials by open circles. The red horizontal dotted line indicates the null effect. The diagonal line shows the meta-regression line with *P* = 0.0008 for the test that the slope is null. There are no indications of residual heterogeneity over the regression line, *P* = 0.9. For the calculations, see Supplementary File 1.

In the 12 trials included in our meta-analysis, baseline LVEF varied from 35 to 66%, but only the Ho trial with HF patients (41) had a baseline LVEF below 48%. The relationship between the effect of vitamin C and the baseline LVEF is plotted in Figure 5 with the cardiac trials indicated by filled circles and the non-cardiac trials by open circles. The evidence for modification of the vitamin C effect by the baseline LVEF is very strong (*P* = 0.0008). There is no evidence of residual heterogeneity around the regression line, which indicates that the meta-regression fully captures the findings of the 12 trials. The slope for all 12 trials crosses the null-effect line at a baseline LVEF of close to 70%. Given that the Ho trial (41) had by far the lowest baseline LVEF level of 35%, and is thereby particularly influential in defining the slope, it was removed in a sensitivity analysis, however, the slope did not change substantially and remained significant (*P* = 0.011). It is worth noting that most of the non-cardiac trials had participants with high baseline LVEF levels, which explains their rather low pooled estimate of effect in Figure 4.

Three before-after trials were excluded from our meta-analysis. The Jensen trial (38) was a study of adult patients with transfusional iron overload. During 12-month iron chelation therapy with desferrioxamine, the LVEF level of the patients significantly decreased. Thereafter, 0.2 g/day of vitamin C was administered in order to increase the efficacy of iron chelation. After 6 months of vitamin C administration, there was a mean increase in LVEF of 7.0% (*P* < 0.01) in 9 patients compared with a LVEF of about 56% before vitamin C; see Supplementary File 2 for the calculation. Because of the continuous gradual decline in LVEF before vitamin C administration, the net effect of vitamin C would be even greater, if the period before vitamin C was adjusted for. In two pediatric trials with T1D patients and healthy controls, Sabri et al. (49) gave 0.25 g/day vitamin C for 1 month. No increase in LVEF was observed from the baseline LVEF of 66% (49). Given the baseline LVEF levels of these three trials, these findings are consistent with the predictions of our meta-regression in Figure 5.

#### Vitamin C and Other Measures of Cardiac Function in the Included Trials

Five of the included publications also reported other outcomes relevant to the topic of this study.

Basili et al. reported that after the percutaneous coronary intervention (PCI), the Thrombolysis in Myocardial Infarction Myocardial Perfusion Grade (TMPG, a categoric coronary flow grading system) was normal in 79% (22/28) of the participants in the vitamin C group, compared with just 39% (11/28) in the placebo group (*P* = 0.01) (43). In addition, after the PCI, the corrected Thrombolysis in Myocardial Infarction frame count (cTFC; a quantitative index for the assessment of myocardial perfusion) was lower, indicating better reperfusion in the vitamin C group (median change −41%) compared with the placebo group (median change −23%; *P* < 0.0001).

Ho found that, compared with the placebo group, the 4-week vitamin C administration increased the distance covered in a 6-min walk test by 26% in the HF patients (*P* < 0.001) and the Minnesota Living with HF questionnaire score by 53% (*P* = 0.01) (41).

Scalzo et al. found that the effect of vitamin C was not significant on left ventricular circumferential and longitudinal strain, which are measures of systolic function, whereas there was a marginally significant effect of vitamin C on the decrease in the post-peak exercise measurement of circumferential strain (*P* = 0.052) (52). In both the healthy and the T2D patients, vitamin C infusion improved diastolic function, estimated by lower values of lateral and septal E:E' (*P* < 0.05 for both) indicating enhanced relaxation. In the mitral valve deceleration time, there was interaction between the vitamin C intervention and the participant group (healthy vs. T2D) (*P* = 0.018), such that vitamin C decreased mitral valve deceleration time in participants with T2D.

Gao et al. studied the same participants before and after hyperoxia. Systolic myocardial velocity was higher during hyperoxia with a vitamin C infusion compared with the control day on which no vitamin C infusion was given (*P* = 0.001) (48). Vitamin C also led to a higher coronary blood velocity during hyperoxia compared with the control day (*P* = 0.005), and a decrease in coronary vascular resistance (*P* = 0.04).

Guan et al. reported that in patients with acute myocardial infarction undergoing direct PCI, intravenous vitamin C did not affect the cardiac index, measured by thermodilution technique at 3–4 weeks after the onset (39).

## Discussion

Textbooks often describe the effect that vitamin C has on wound healing, explaining the effect through the role of vitamin C in collagen metabolism. However, this is a simplistic view of the physiological functions of the vitamin. Biochemistry of vitamin C is complex, extending from several cofactor roles in diverse parts of metabolism to non-specific antioxidant effects, and further to wide-ranging epigenetic effects (22–26). The numerous biochemical effects translate to diverse changes at the clinical level. Vitamin C deficiency is associated with many symptoms characteristic of HF (1–15).

Compared with healthy people, patients with HF have lower vitamin C levels, which are not explained by differences in dietary intake levels (57–59). More severe HF seems to be associated with lower plasma vitamin C levels (57, 58). The apparent depletion of vitamin C in HF may be explained by increased metabolic consumption due to the oxidative stress associated with HF (60). In the early literature, vitamin C was suggested for treatment of HF (61–64), yet interest in the topic waned. It seems probable that the early findings were ignored because of wide-spread bias against vitamin C having effects other than treating and preventing scurvy (65).

Decreased plasma vitamin C levels have also been reported after cardiac surgery in parallel with increases in the oxidized forms of the vitamin, i.e., dehydroascorbate and ascorbate free radical (66–72). Thus, if vitamin C has an effect on cardiac function it is plausible that the decreased plasma vitamin C levels may contribute to the postoperative compromise in myocardial function after cardiac surgery.

In this systematic review, six cardiac trials were included. One of them was a 28-day oral vitamin C supplementation trial in patients with HF, whereas the others were 1-day intravenous vitamin C trials in patients who had undergone cardiac surgery or PCI. In the cardiac trials, vitamin C increased the LVEF levels on average by 12%. We also pooled six non-cardiac trials with diverse clinical contexts. In these trials vitamin C increased the LVEF levels on average by 5%. The statistically highly significant effect of vitamin C in both the cardiac and non-cardiac trials provides strong evidence that in some contexts vitamin C can influence the mechanical functions of the heart. Three further before-after trials were consistent with our meta-regression analysis.

We did not demonstrate a dose response on the size of the effect. There was also no difference between oral and intravenous administration, which suggests that intravenous administration is not necessarily needed, despite the fact that this was the mode used in most studies (Table 1). However, the included trials were small and examined mostly ambulant patients, or patients undergoing elective cardiac surgery, and not patients with more severe disease with greater oxidative stress. Therefore, our comparison of oral and intravenous administration, while indicating that in some contexts oral vitamin C is effective, is not definitive and should not be generalized widely.

We found a strong negative association between the size of the vitamin C effect and baseline LVEF (Figure 4). However, only a single trial, which investigated HF patients (41), had baseline LVEF levels below 48% and that trial has the greatest weight in the linear regression. Nevertheless, the association remained significant even when the HF trial was removed. This modification of the vitamin C effect by baseline LVEF should be examined in further trials. Previously, the severity of disease was found to modify the effect of vitamin C on intubation time (73), the effect of vitamin C on FEV_1_ decline as a result of exercise tests (74), and the effect of vitamin C on the duration of COVID-19 in outpatients (65, 75).

A few of the included trials reported effects of vitamin C on measures of cardiac physiology other than LVEF and found that vitamin C increased cardiac perfusion after PCI (43, 44), improved diastolic function in an exercise test (52), and increased systolic myocardial velocity during hyperoxia (48). In addition to the included trials, a few other trials have reported that vitamin C had an effect on the mechanical function of the heart (76–79).

Two cohort studies examined the effects of dietary vitamin C intake in HF patients who had average LVEF levels of 34% (80, 81). In both studies, higher dietary vitamin C intake was associated with a lower rate of cardiac events during follow-up. In addition, higher vitamin C intake was associated with a better health related quality of life (81). Residual confounding is a potential concern in cohort studies. However, in the randomized cross-over trial with HF patients included in our analysis, Ho found significant benefit from vitamin C administration on health-related quality of life and on the 6-min walk test (41). One of the trials included in our meta-analysis reported both a significant increase in LVEF and a significant decrease in ICU stay in the vitamin C participants (53), and another reported a significant increase in LVEF and a significant decrease in intubation time (51). It is plausible that the effect of vitamin C on the mechanical function of the heart is one of the explanations for the benefits seen in some ICU patients (65, 73, 82–84).

No effect of vitamin C on cardiovascular events was found in the Physicians' Health Study II (PHS-II) (85), in which 14,641 participants received 0.5 g/day of vitamin C for 8 years. However, the PHS-II trial recruited physicians, a group of extremely health-literate professionals who are not representative of the average population in terms of health-related lifestyles. Thus, the participants in the PHS-II trial were very different from the participants in the trials we included in our meta-analysis (Table 1) and so the PHS-II trial should not be considered a relevant comparison for the current meta-analysis.

It is plausible that increased intake of vitamin C may have no beneficial effect for well-nourished healthy people, but higher doses may have effects for people under heavy physiological stress, for example during cardiac surgery, or for people with HF. The possible effects of vitamin C for patients under heavy physiological stress is supported by controlled trials which found vitamin C to be beneficial for ICU patients (65, 73, 82–84), patients undergoing cardiac operations (28), patients with exercise-induced bronchoconstriction (74, 86), and patients with short-term respiratory symptoms induced by heavy physical activity (87–89).

One potential concern with meta-analyses is publication bias. Most of the included trials did not mention the findings on vitamin C and LVEF in their abstracts (39, 43, 44, 46–50, 52), and the Ho study was published only as a monograph (41). This indicates that the specific findings on LVEF were not the primary reason for publication. Furthermore, it is highly unlikely that publication bias could generate the association shown in Figure 5.

The method of allocation and the level of blinding during controlled trials are important issues. There was no risk of bias associated with these issues for most of the included trials (Figure 2). Nevertheless, even when the analysis was restricted to the four cardiac trials for which there were no methodological concerns, the evidence indicating a benefit from vitamin C was very strong.

In conclusion, in this meta-analysis, vitamin C increased LVEF in both cardiac and non-cardiac patients, with a strong negative association between the size of the vitamin C effect and the baseline LVEF. Further research on vitamin C and HF should be carried out. The most informative approach would be to investigate patients who have low LVEF together with low vitamin C intake or plasma levels, and to compare different dosages and different routes of administration. If an effect of vitamin C is demonstrated in such a setting, it would be reasonable thereafter to examine patients with higher LVEF levels or higher plasma vitamin C levels to determine the relationship between baseline characteristics and the effects of vitamin C.

## Methods

We searched for controlled trials that reported LVEF levels in parallel vitamin C and control groups, in cross-over trials (separate vitamin C and control periods for the same participants), and before-after studies. We included trials in which the administration of vitamin C was the only difference between the study groups or periods. We did not limit our search to randomized trials and did not require placebo control. We included all doses, all routes of administration and all durations of vitamin C administration.

We searched MEDLINE, EMBASE, and the Cochrane Central Register of Controlled Trials with the search phrases described in Supplementary File 1. Two authors (HH and AM) independently screened the titles and abstracts and identified potentially relevant trials. Discrepancies between reviewers were resolved by discussion. We also perused the reference lists of the selected trials and relevant reviews. We identified 16 publications reporting on 15 distinct trials that satisfied our selection criteria (38–53) (Figure 1, Table 1). One author (HH) extracted study characteristics and outcomes from the trial reports and entered the data in Supplementary Tables S1, S2 and in a spreadsheet, which are available in Supplementary Files 1, 2. All authors checked the data entered against the original trial reports and all authors assessed the methodological quality of the trials (Figure 2, Supplementary Table S1). We contacted several authors to ask for details of their trials, but only three authors responded; see Supplementary Table S1.

The primary outcome in this analysis was the change in LVEF after the initiation of vitamin C administration. For most trials, we used variance analysis to calculate the *P*-value for the interaction between time and vitamin C treatment; see Supplementary File 1 for the formula to calculate the F-test from the reported mean and standard deviation (SD) values of LVEF, and Supplementary File 2 for the calculations. Thereafter we used the *P*-values to calculate comparable standard error (SE) values for the differences between the vitamin C and control observations. For the Jensen trial, we measured the LVEF changes from the 6-month time point of their figure 4A (38); see Supplementary File 2.

As secondary outcomes, we collected other measures of cardiac function from the included trial reports. The findings for the secondary outcomes are described in the Results section, but we did not construct forest plots for any of them because of their heterogeneity and low number.

In our analysis of the changes in LVEF, we used the relative scale, which has been shown to be superior in the analysis of continuous outcomes, because it adjusts for baseline variations and frequently leads to less heterogeneity (54–56). An illustration of the measurement of the vitamin C effect on LVEF is shown in Figure 3 by using the Basili trial (43) as an example. Our analysis of the changes in LVEF is closely analogous to a previous analysis of changes in FEV_1_ (54). In both cases the original observations are expressed as a percentage change, and the relative change caused by the treatment is calculated as a percentage effect of the percentages (Figure 3). We pooled the included trials with the *metagen* function of the R package *meta* (90–92), using the inverse variance, fixed effect options. For the meta-regression of the vitamin C effect on baseline LVEF, we used the *metareg* function of the *meta* package.

Although the Cochran Q test has been criticized (93), in the absence of a suitable alternative we used it to assess statistical heterogeneity among the trials in the meta-analysis, but we did not calculate the *I*^2^ statistic. Our calculations are described in Supplementary Files 1, 2.

## Data Availability Statement

The original contributions presented in the study are included in the article/Supplementary Material. Further inquiries can be directed to the corresponding author/s.

## Author Contributions

HH planned the study, extracted the data and entered the data into a spreadsheet, assessed the quality of the included trials, carried out the statistical analysis, and wrote the draft manuscript. EC and AM checked that the entered data were consistent with the published data, independently assessed the quality of the included trials, and participated in the critical revision of the manuscript. All authors read and approved the final manuscript.

## Conflict of Interest

The authors declare that the research was conducted in the absence of any commercial or financial relationships that could be construed as a potential conflict of interest.

## Publisher's Note

All claims expressed in this article are solely those of the authors and do not necessarily represent those of their affiliated organizations, or those of the publisher, the editors and the reviewers. Any product that may be evaluated in this article, or claim that may be made by its manufacturer, is not guaranteed or endorsed by the publisher.

## Supplementary Material

Supplementary File 1 Detailed descriptions of the included trials and statistical calculations.

Supplementary File 2 Spreadsheet with the calculations of the results.
