# Supplementary material for: Vitamin C May Improve Left Ventricular Ejection Fraction: A Meta-Analysis
Source: Front Cardiovasc Med. 2022 Feb 25;9:789729. doi: 10.3389/fcvm.2022.789729 (PMC8913583; doi:10.3389/fcvm.2022.789729)
Supplement: Supplementary File 1 — Detailed descriptions of the included trials and statistical calculations. [file Data_Sheet_1.PDF]

Vitamin C may improve left ventricular ejection fraction: a meta-analysis

Supplementary Material

Harri Hemilä, Elizabeth Chalker, Angelique de Man

Harri Hemilä, MD, PhD
Department of Public Health,
University of Helsinki, POB 41,
Helsinki, FI-00014, FINLAND.
E-mail: harri.hemila@helsinki.fi
https://www.mv.helsinki.fi/home/hemila

ver 2022-1-10

Contents **Page**

Search terms for the data base searches 2

Table S1: Detailed description of the included trials 3

Table S2: Extraction of the results of the included trials 23

Figure S1: Comparison of vitamin C and control arms 24

Calculation of the P-value for the interaction between vitamin C and time in the LVEF trials 25

Printouts of statistical calculations 28

Search terms for the data base searches

PUBMED 2021-7-22

("ascorbic acid"[MeSH] OR "vitamin c"[tiab]) AND

("ejection fraction"[tiab] OR ("heart failure"[MeSH] OR "heart failure"[tiab]) OR ("heart function tests"[MeSH] OR "cardiac function"[tiab] OR "heart function"[tiab]) OR ("stroke volume"[MeSH] OR "stroke volume"[tiab]) OR ("myocardial reperfusion injury"[MeSH] OR "myocardial injury"[tiab] OR "reperfusion injury"[tiab]) OR ("percutaneous coronary intervention"[MeSH] OR "percutaneous coronary intervention"[tiab]) OR ("cardiac surgical procedures"[MeSH] OR "cardiac surgery"[tiab]) OR ("cardiopulmonary bypass"[MeSH] OR "cardiopulmonary bypass"[tiab]))

809 records > **10 relevant**

EMBASE 2021-7-22

('ascorbic acid'/exp OR 'ascorbic acid') AND ('ejection fraction'/exp OR 'ejection fraction' OR 'heart failure'/exp OR 'heart failure' OR 'heart function test'/exp OR 'heart function test' OR 'stroke volume'/exp OR 'stroke volume' OR 'myocardial reperfusion injury'/exp OR 'myocardial reperfusion injury' OR 'percutaneous coronary intervention'/exp OR 'percutaneous coronary intervention' OR 'cardiac surgery'/exp OR 'cardiac surgery' OR 'cardiopulmonary bypass'/exp OR 'cardiopulmonary bypass')

2028 records > **10 relevant**

CENTRAL Cochrane Library 2021-6-28

("vitamin C" OR ascorb*) AND

("heart failure" OR "ejection fraction" OR "cardiopulmonary bypass" OR "percutaneous coronary intervention")

138 records > **7 relevant**

OTHER SOURCES

5 reports

Table S1: Detailed description of the included trials

Basili 2010

**Methods**	Randomized double-blind placebo-controlled trial, Sep 2007 to Dec 2008.<br>https://doi.org/10.1016/j.jcin.2009.10.025<br>https://www.ncbi.nlm.nih.gov/pubmed/20170881<br>**A related publication of the same RCT, with LVEF results** (p 389)(Pignatelli 2011):<br>https://doi.org/10.1111/j.1755-5922.2010.00168.x<br>https://www.ncbi.nlm.nih.gov/pubmed/20629665<br>**A third publication of the same RCT but no LVEF data**<br>https://doi.org/10.1161/atvbaha.111.227959<br>https://pubmed.ncbi.nlm.nih.gov/21636808/
**Participants**	Italy, elective PCI patients, 47 M / 9 F, mean age: 67 y (SD 9), 28 vit C / 28 placebo.<br>**Inclusion:** ≥ 18 years of age, clinically stable class I or II effort angina pectoris according to the Canadian Cardiovascular Society criteria, a positive functional study for myocardial ischemia and a single de novo lesion in a native coronary artery that was scheduled for elective PCI.<br>**Exclusion:** Contraindication to aspirin or clopidogrel, previous myocardial infarction, multivessel interventions, graft vessel disease, low platelet count, history of bleeding diathesis and renal dysfunction.
**Interventions**	**Vitamin C:** "1 g of vitamin C ... diluted in 250 ml of isotonic saline infused at 16.6 mg/min, 4.2 ml/min, over 1 h before PCI" (p 223).<br>The parallel publication (Pignatelli 2011) described the dosage somewhat differently as:<br>"intravenous infusion of ascorbic acid (1 g/L at 24 mg/min)" (p 387).<br>**Control group:** "placebo (isotonic saline infused at 4.2 ml/min over 1 h before PCI)" (p 223).
**Outcomes**	LVEF<br><u>Secondary outcomes:</u><br>cTFC (corrected Thrombolysis In Myocardial Infarction frame count; measures coronary flow)<br>TMPG (Thrombolysis In Myocardial Infarction myocardial perfusion grade; measures microvascular perfusion)
**Method and timing to measure LVEF**	Echocardiography with calculation of LVEF by the biplane Simpson's rule, based on "LVEF ... as recommended by the American Society of Echocardiography at baseline and 72 h after PCI" (p 223).
**Source of data for LVEF**	Text in left-hand column p 225.
**LVEF at baseline**	Vit C 52.3%, Placebo 53.7%
**Notes**	We were able to contact Dr. Francesco Violi by email on 2020-3-13 and 2020-3-21 and we received further information about the methods, see below.

Risk of bias table

Bias	Authors' judgement	Support for judgement
Random sequence generation (selection bias)	Low risk	"randomly assigned in a 1:1 manner" (p 223).<br>"… those patients who continue to meet eligibility criteria were randomized to placebo or ascorbic acid administration and received a unique study identification code. Thus, after baseline collection of blood samples a sealed, opaque envelopes containing a computer-generated random sequence were used for randomization to an intravenous infusion of ascorbic acid ... or placebo (saline solution)" (p 387 Pignatelli 2011).<br>Groups were balanced for eg. age, hypertension, dyslipidemia, smoking, diabetes mellitus, drug usage (Table 1).
Allocation concealment (selection bias)	Low risk	"those patients who continued to meet eligibility criteria were randomized to placebo or ascorbic acid administration and received a unique study identification code" (p 223 Basili).<br>"Thus, after baseline collection of blood samples a sealed, opaque envelopes containing a computer-generated random sequence were used for randomization to an intravenous infusion of ascorbic acid ... or placebo (saline solution)" (p 387 Pignatelli 2011).
Blinding of participants and personnel (performance bias)	Low risk	The randomization list was unveiled after that the analytical phase was terminated" (p 387 Pignatelli 2011).
Blinding of outcome assessment (detection bias)	Low risk	"To ensure blind analysis, cardiologists sent tube identified by numerical code to the laboratory where biologists perform analytical tests. The randomization list was unveiled after that the analytical phase was terminated" (p 387 Pignatelli 2011).<br>"each lab investigation was blinded, echography included"<br>(email from Francesco Violi 2020-3-21).
Incomplete outcome data (attrition bias)	Low risk	No description in the papers, but "We did not have any drop-out"<br>(email from Francesco Violi 2020-3-13).

Emadi 2019

**Methods**	Randomized double-blind placebo-controlled trial<br>https://doi.org/10.21470/1678-9741-2018-0312<br>https://www.ncbi.nlm.nih.gov/pmc/articles/PMC6852463<br>https://www.ncbi.nlm.nih.gov/pubmed/31719005
**Participants**	Iran, elective CABG patients, 32 M / 18 F; mean age 62 y (SD 8), 25 vit C / 25 placebo. The N was concluded from Table 1.<br>**Inclusion:** patients who were referred to hospital for undergoing CABG.<br>**Exclusion:** History of arrhythmias, EF < 30%, severe renal or hepatic failure, pacemaker, antiarrhythmic drugs and digoxin, AV-block or bradycardia, history of recent myocardial infarction, high initial troponin I level, and history of redo or complex cardiac surgery.
**Interventions**	**Vitamin C:** "5 g of intravenous vitamin C before induction of anesthesia and 5 g of vitamin C in the cardioplegic solution" (p 518).<br>**Control group:** "the same amount of placebo (normal saline)" (p 518).
**Outcomes**	LVEF
**Method and timing to measure LVEF**	"LVEF was measured by transthoracic echocardiography" (p 518). Time points were preoperation vs 3 days postoperation.<br>The method of LVEF measurement by echocardiography was not described. The methods of LVEF measurement with echocardiography differ with regard to the type of echocardiographic image used (M-mode, 2D or 3D), the measurements needed and the equations/assumptions used to determine LV volumes.
**Source of data for LVEF**	Table 1 reports the pre-operative and Table 4 reports the post-operative LVEF levels.
**LVEF at baseline**	Vit C 56.29%, Placebo 56.50%
**Notes**	We tried to contact Dr. Allahyari by email on 2020-3-13 and 2020-3-20 but we did not receive any responses.

Risk of bias table

Bias	Authors' judgement	Support for judgement
Random sequence generation (selection bias)	Low risk	"randomized clinical trial study ... Sample selection was done using a block randomization method " (p 518).<br>Groups were balanced (Table 1) for age, sex, height, weight, blood pressure, LVEF.
Allocation concealment (selection bias)	Low risk	"double-blind" (p 518) indicates that all people, patients and researchers were unaware of treatment from the very beginning until the very end of the trial.
Blinding of participants and personnel (performance bias)	Low risk	See above
Blinding of outcome assessment (detection bias)	Low risk	See above
Incomplete outcome data (attrition bias)	Low risk	No flow diagram, no description of whether or not there were dropouts. However, the equal size of the reported groups suggests that the groups may have been the same size at the start as indicated by the description of "a block randomization method". The study was short.

Fernhall 2010

**Methods**	Randomized double-blind placebo-controlled trial<br>https://doi.org/10.1177/1358863X11404940 (Fahs 2011)<br>https://doi.org/10.1007/s00421-011-2033-x (Fernhall 2012).<br>https://www.ahajournals.org/doi/10.1161/circ.122.suppl_21.A11015 (Fernhall 2010 abstract).
**Participants**	USA, healthy firefighters, 69 M / 0 F; mean age 28 y (18-64), 34 vit C / 35 placebo.
**Interventions**	1 h before the pre-firefighting data collection, all firefighters ate a standardized meal. With this meal, each participant consumed either 2 g vitamin C or identical placebo (p 114 in Fahs 2011). "The vitamin C was delivered with the meal as a tablet – or placebo with an identical looking tablet – the tablets were consumed immediately following the meal" (email Fernhall 2021-3-16).<br>"An oral dose of 2 g of vitamin C 1 hour prior to firefighting activities" (p 115 in Fahs 2011).<br>"identical placebo" (p 114 in Fahs 2011) "the Vitamin C tablets were identical in size, shape and color to the placebo tablets" (email Fernhall 2021-3-16).
**Outcomes**	LVEF
**Method and timing to measure LVEF**	"high definition ultrasound. Standard M-mode and B-mode imaging coupled with tissue Doppler imaging (TDI) at the mitral annulus" (Fernhall 2010)
**Source of data for LVEF**	Table in the abstract Fernhall (2010).
**LVEF at baseline**	Vit C 58%, Placebo 61%
**Notes**	We contacted Dr. Bo Fernhall by email and received further information about the methods on 2021-3-16

Risk of bias table

Bias	Authors' judgement	Support for judgement
Random sequence generation (selection bias)	Low risk	"male firefighters randomly assigned" (Fernhall 2010). "The randomization was done using a computer program by our biostatistician who never met a participant" (email Fernhall 2021-3-16).
Allocation concealment (selection bias)	Low risk	"double blind" (Fernhall 2010). "All activities were done under blinding, we only unblinded the study after all data were collected and all of the statistics had been completed" (email Fernhall 2021-3-16).
Blinding of participants and personnel (performance bias)	Low risk	see above
Blinding of outcome assessment (detection bias)	Low risk	see above
Incomplete outcome data (attrition bias)	Low risk	"There were no drop-outs – unusual for most studies but not in this type of firefighter work – the firefighters were incredibly motivated to participate" (email Fernhall 2021-3-16).

Gao 2012

**Methods**	Cross-over trial; first control and then vitamin C<br>https://doi.org/10.1007/s00421-011-1997-x<br>https://www.ncbi.nlm.nih.gov/pmc/articles/PMC3281262<br>https://www.ncbi.nlm.nih.gov/pubmed/21584682
**Participants**	USA, healthy, 4 M / 4 F, mean age 26.5 y, same participants for both control and vitamin C periods. In both periods participants were exposed to hyperoxic challenge.
**Interventions**	Vitamin C was administered before exposure to hyperoxic challenge.<br>**Vitamin C day:** "On the other day, subjects breathed room air for 10 min, then received an intravenous infusion of 3.0 g of vitamin C over 15 min, and then were given 100% oxygen to breath for 10 min. The hyperoxia began 10 min into the vitamin C infusion" (p 484).<br>**Control day:** no saline infusion. "In one hyperoxic challenge, subjects breathed room air for 10 min, and then breathed 100% oxygen via a plastic facemask for 10 min" (p 484).
**Outcomes**	LVEF<br>Secondary outcomes:<br>Sm (systolic myocardial velocity)<br>CBV (coronary blood velocity)<br>CVR (coronary vascular resistance)
**Method and timing to measure LVEF**	Echocardiography with calculation of LVEF using Simpson's biplane rule (p 484)
**Source of data for LVEF**	Table 2
**LVEF at baseline**	Vit C 58.63%, Control 60.38%
**Notes**	We contacted Dr. Sinoway by email on 2021-3-16 but did not receive a response

Risk of bias table

Bias	Authors' judgement	Support for judgement
Random sequence generation (selection bias)	Low risk	"All subjects underwent two hyperoxic challenges (oxygen alone and oxygen with an infusion of vitamin C), which were performed on two separate days" (p 484).
Allocation concealment (selection bias)	Unclear risk	No description
Blinding of participants and personnel (performance bias)	Unclear risk	No description
Blinding of outcome assessment (detection bias)	Unclear risk	No description
Incomplete outcome data (attrition bias)	Low risk	Paired data

Glavas 2009

**Methods**	Randomized cross-over trial<br>https://doi.org/10.1111/j.1475-097x.2008.00845.x
**Participants**	Croatia, divers, 8 M / 0 F, mean age 37 y (SD 13)
**Interventions**	A single dose of vitamin C (10 mg/kg body weight) dissolved in orange juice 3 h before dive. Average BMI was 26.3 and average height was 1.82 m, which indicates that average weight was about 87 kg. Thus, vitamin C dose was about 0.87 g/day, which we round to 1 g/day in our Table 1.<br>Placebo was 200 ml of orange juice
**Outcomes**	LVEF
**Method and timing to measure LVEF**	Two dimensional echocardiographic studies were performed. The cross-sectional axis of the LV at the papillary muscle tip level was measured. Measurements of LV cavity dimensions were carried out at the end-diastolic and end-systolic period. Three consecutive cardiac cycles were measured and average values were obtained. All measurements were made according to American Society of Echocardiography (Sahn et al., 1978). Ejection fraction (EF) of the left ventricle was calculated from the following equation: EF(%) = (EDV – ESV)/EDV. (p 102).<br>"Two hours before the dive and approximately 40 min after surfacing the subjects were investigated by ultrasound." (p 101).
**Source of data for LVEF**	Table 1
**LVEF at baseline**	Vit C 65.8%, Placebo 66.6%
**Notes**	

Risk of bias table

Bias	Authors' judgement	Support for judgement
Random sequence generation (selection bias)	Low risk	"crossover design in which subjects were randomly assigned" "The study was conducted in a double-blind crossover design in which subjects were randomly assigned to either placebo, vitamin C or a combination of BH4 and vitamin C" (p 102)<br>"The divers performed three dives, one as control with placebo; second with vitamin C ... Dives for each participant were randomly assigned." (p 101)<br>"The randomization was performed by the pharmacist, who was not involved in data analysis" (p 102)
Allocation concealment (selection bias)	Low risk	See above
Blinding of participants and personnel (performance bias)	Low risk	See above
Blinding of outcome assessment (detection bias)	Low risk	See above and "The investigators (DG, AO, DB) who performed the measurements were blinded to the group and treatment assignment" (p 102)
Incomplete outcome data (attrition bias)	Low risk	Paired data

Guan 1999

**Methods**	Randomized trial<br>https://doi.org/10.1253/jcj.63.924<br>https://www.ncbi.nlm.nih.gov/pubmed/10614835
**Participants**	Japan, patients with AMI, 14 M / 7 F, mean age 65 y (SD 12), 10 vit C / 11 placebo.<br>**Inclusion:** "Patients with AMI [acute myocardial infarction] ... All patients had typical chest pain with ST-segment elevation on their initial electrocardiogram" (p 924)
**Interventions**	Guan described that "...vitamin C, which was intravenously infused at an initial dose of 2000mg followed by a constant infusion at 20 mg/min ... The constant infusion of vitamin C was continued throughout the study period" (p 924).<br>Guan does not describe the duration of the study period. A PTCA usually lasts about 50 minutes and in the study urine samples were collected for 150 min after PTCA. Thereby we estimate that the dose of vitamin C was about 200 * 20 = 4000 mg after the initial dose. Thereby we estimate that the total dose of vitamin C was about 6 g.<br>Group 1 was the control, but no description of possible placebo infusion
**Outcomes**	LVEF<br>Secondary outcomes:<br>Cardiac index (measured by thermodilution procedure at admission and after 3-4 weeks (p 925 right bottom).<br>However, there is no description of the thermodilution procedure in the methods section.
**Method and timing to measure LVEF**	"left ventriculography" (p 925)
**Source of data for LVEF**	Text section of Results (p 925 right-bottom).
**LVEF at baseline**	Vit C 51%, Control 49%
**Notes**	

Risk of bias table

Bias	Authors' judgement	Support for judgement
Random sequence generation (selection bias)	Low risk	"randomly divided into 2 groups" (p 924).<br>Table 1 shows that several relevant baseline variables were closely balanced between the treatment groups (p 925).<br>Although the details of randomization are not described, we do not consider that there is high risk of bias in the negative finding, as several baseline variables were balanced.
Allocation concealment (selection bias)	Low risk	No description. However, the finding is negative and we do not consider that it is likely that a negative finding could be explained by poor blinding at the stage of allocation.
Blinding of participants and personnel (performance bias)	Low risk	No description. However, the finding is negative and we do not consider that it is likely that a negative finding could be explained by poor blinding after allocation.
Blinding of outcome assessment (detection bias)	Low risk	See above
Incomplete outcome data (attrition bias)	Low risk	See above

Ho 2007

**Methods**	Randomized cross-over trial<br>http://rave.ohiolink.edu/etdc/view?acc_num=case1157980994
**Participants**	Taiwan, patients with HF, 24 M / 13 F, mean age 69 y (SD 16)<br>**Inclusion:** (a) documented diagnosis of HF for longer than 3 months; (b) NYHA functional II and III; (c) between 40 and 80 years of age, because most patients under 40 who suffer from HF originate from cardiomyopathy rather than ischemia; (d) literate in Chinese; and, (e) no reports of mental impairment (p 58).<br>**Exclusion:** (a) being allergic to ascorbic acid; (b) regularly using a vitamin C supplement, for the principal investigator will hardly be able to identify the effects of antioxidant supplementation; (c) having serum creatinine levels greater than 3.5 mg/dl; (d) being diagnosed with severe vascular disease, uncontrolled hypertension, a history of cardiac arrest or life-threatening arrhythmia within the preceding three months; (e) having experienced an acute myocardial infarction, acute coronary syndrome, or stroke within the preceding three months; (f) having had previous coronary artery bypass grafting surgery (CABG) or valve replacement within the preceding three months or the likelihood of requiring such a procedure during the study period; (g) having a left ventricular assist device or automatic implanted cardiovertor-defibrillator device; (h) having mechanical ventilator support; and, (i) women if they are pregnant, nursing, or of childbearing age and not using an effective method of contraception (p 58).
**Interventions**	"Group I ... received vitamin C 2000 mg twice daily (4000 mg daily) for four weeks, followed by a placebo for six weeks" (p 79).<br>"Group II ... received a placebo for six weeks, followed by vitamin C 2000 mg twice daily (4000 mg daily) for four weeks" (p 79).<br>"the PI gave them vitamin C or placebo in a bottle" (p 77) implies that tablets were used.
**Outcomes**	LVEF<br>Secondary outcomes:<br>Minnesota Living with Heart Failure Questionnaire (MLHFQ)<br>Exercise capacity using 6-minute distance walk test (6MWT)
**Method and timing to measure LVEF**	"two-dimensional echocardiography" (p 67).<br>"LVEF and other parameters were calculated through a standard echocardiogram using Simpson's method" (p 68)
**Source of data for LVEF**	Table 4-18 (p 101): The vitamin C and placebo period means and SDs were pooled, see Supplementary file 2.
**LVEF at baseline**	Vit C 34%, Placebo 36%
**Notes**	We tried to contact Dr. Ho by email on 2020-4-14 and 2020-4-18 but we did not receive a response.

Risk of bias table

Bias	Authors' judgement	Support for judgement
Random sequence generation (selection bias)	Low risk	"Permuted block randomization was performed using the SAS 9.0 software program... blocks of 6 subjects per stratum were used. Eligible subjects were allocated to Group I (AB) or Group II (BA) based on the SAS generating sequence" (p 79).<br>The participants were their own controls and therefore systematic baseline differences are unlikely.
Allocation concealment (selection bias)	Low risk	See above
Blinding of participants and personnel (performance bias)	Low risk	"the PI gave them vitamin C or placebo in a bottle" (p 77) implicitly suggests that the participants were blinded.
Blinding of outcome assessment (detection bias)	Unclear risk	No description
Incomplete outcome data (attrition bias)	Low risk	Paired data. "3 subjects (2 males and 1 female) had to be dropped from this study due to personal reasons" (p 82). Each participant would have participated in both a placebo and a vitamin C period, thus there cannot be bias between periods if a person drops out from both periods.

Jensen 1997

**Methods**	Before-after, self-control<br>https://doi.org/10.1111/j.1600-0609.1997.tb00981.x<br>https://www.ncbi.nlm.nih.gov/pubmed/9338620
**Participants**	Denmark, patients with iron load, 1 M / 8 F, mean age 52 y (37-67)<br>**Inclusion:** Iron-loaded adult nonthalassemic patients treated with desferriosamine before vitamin C.
**Interventions**	"Vitamin C (200 mg OD) was given in order to increase the efficacy of iron chelation. In order to prevent a too strong (and potentially harmful) iron mobilization by vitamin C during the first period of time of iron chelation, vitamin C supplementation was not started until after 12 months of iron chelation" (p 223).
**Outcomes**	LVEF, baseline LVEF level when on desferriosamine alone, and vitamin C effect after 6 months supplementation
**Method and timing to measure LVEF**	"LVEF was assessed at rest by MUGA" (p 223)
**Source of data for LVEF**	Fig 4a: at the time point of 6 months, individual patient data for 9 participants was measured from the figure and t-test for the change from the start of vitamin C was calculated, see Supplementary file 2.
**LVEF at baseline**	55.9% (p 227)
**Notes**	Excluded from our meta-analysis because trial did not have an explicit control group or control period. Before the vitamin C period there was a 1-year period without vitamin C and there was continuous decline in LVEF levels during that period, but we do not consider that period suitable as a control period in our analysis.<br>"After vitamin C supplementation was started, the mean LVEF increased progressively (Fig. 1b) from 55.9% (5.3) at the start to 65.3% (9.8) after 12 months (p=0.01)" (p 227)

Risk of bias table

Bias	Authors' judgement	Support for judgement
Random sequence generation (selection bias)	Unclear risk	Participants were controls for themselves
Allocation concealment (selection bias)	Unclear risk	see above
Blinding of participants and personnel (performance bias)	Unclear risk	It seems evident that participants knew that they were given vitamin C. However, we do not consider it likely that knowledge of vitamin C by the patient caused high risk of bias in the LVEF measurement. As to outcome assessment, see below.
Blinding of outcome assessment (detection bias)	Low risk	"All radionuclide recordings were analysed by the same experienced observer to eliminate any interobserver variation. The observer did not know what treatment the patient was receiving" (p 223).
Incomplete outcome data (attrition bias)	Low risk	Vitamin C was discontinued for 1 of 9 patients: "Patient 1 had myelodysplastic syndrome (RARS). Due to coexisting ischaemic heart disease, the Hb concentration was maintained above 11 g/dl requiring a high measure of blood transfusion (8 blood units per month). His LVEF decreased slightly but progressively during the first 9 months of iron chelation. In order to improve his progressively decreasing LVEF due to the insufficient iron depletion as measured by unchanged liver iron concentration, the patient began oral vitamin C supplementation (200 mg OD). After approximately 2 months he developed progressive right and left heart failure (LVEF 25%). Echocardiography revealed severe impairment of the systolic ventricular function, but extensive investigation did not reveal the reason for this deterioration. In order to exclude a possible cardiotoxic effect of the iron chelation therapy, Both DFO and vitamin C were discontinued for a month and treatment with digoxin, amiodarone and diuretics was started and within 1 month the cardiac failure reversed The patient was again given DFO (2 g by pump) and after one month of iron chelation MUGA revealed an almost normalized LVEF (49%)" (p 227).

Oktar 2001

**Methods**	Controlled trial<br>https://doi.org/10.1080/003655101753267982<br>https://www.ncbi.nlm.nih.gov/pubmed/11768322
**Participants**	Turkey, CABG patients, 20 M / 4 F, mean age 56 y (SD 9), 12 vit C / 12 control.<br>**Inclusion:** "patients undergoing coronary artery cardiopulmonary bypass bypass grafting" (p 622).<br>**Exclusion:** No description.
**Interventions**	**Vitamin C:** "Group II received 4 g of ascorbic acid intravenously just before the induction of anaesthesia" (p 622).<br>**Control group:** "Group IV served as the control group " (p 622). No description of the treatment in the control group.
**Outcomes**	LVEF
**Method and timing to measure LVEF**	"Echocardiography was performed for all patients preoperatively and on the same days postoperatively, and EF was used as a parameter in the assessment of myocardial function" (p 623). Time points were preoperation vs 3 and 7 days postoperation. In our analysis we use data from the 7 days time point.<br>The researchers also performed MUGA scans. This method can also be used to estimate LVEF. However, the authors did not report LVEF determined by MUGA scan, only peak ejection rate.
**Source of data for LVEF**	Table III: Groups II (Vitamin C prior to induction) and IV (Control); Comparison of Postop. 7th day vs. Preoperative data.
**LVEF at baseline**	Vit C 59%, Control 64%
**Notes**	Oktar had two vitamin C groups. Group II was administered 4 g vitamin C just before the induction of anaesthesia, whereas Group III was administered 4 g vitamin C in the cardioplegic solutions. Our analysis in Figure 3 uses the Group II findings since vitamin C was given earlier.<br>We tried to contact Dr. Oktar by email on 2020-3-13 and 2020-3-20 but we did not receive any responses.

Risk of bias table

Bias	Authors' judgement	Support for judgement
Random sequence generation (selection bias)	Unclear risk	Groups were balanced (Table I) for eg age, sex, NYHA class, LVEF but there was no description of allocation method.
Allocation concealment (selection bias)	Unclear risk	No description
Blinding of participants and personnel (performance bias)	Unclear risk	No description
Blinding of outcome assessment (detection bias)	Unclear risk	No description
Incomplete outcome data (attrition bias)	Unclear risk	No flow diagram. No description of potential drop outs. However, the identical size in the 4 groups suggests that there may have been no drop-outs in the short trial.

Sabri 2014 healthy

**Methods**	Before-after, self-control<br>https://www.ncbi.nlm.nih.gov/pmc/articles/PMC4258680/
**Participants**	Iran, 19 healthy children, 10 M / 9 F, age 3-18 y<br>**Inclusions:** Non-smokers, non-pregnant and no history of systemic disease<br>**Exclusions:** Those with a history of recent use of vitamin C, non cooperation and diagnosis of new disorders and any type of congenital heart disease, which was found during echocardiography.
**Interventions**	0.25 g/day vitamin C for 1 month
**Outcomes**	LVEF
**Method and timing to measure LVEF**	no description
**Source of data for LVEF**	Table 2
**LVEF at baseline**	64.44%
**Notes**	We contacted Dr. Tavana (second author) by email on 2021-3-11 and 2021-8-1 but we have not received a response.

Risk of bias table

Bias	Authors' judgement	Support for judgement
Random sequence generation (selection bias)	Unclear risk	Participants were controls for themselves
Allocation concealment (selection bias)	Unclear risk	Participants were controls for themselves
Blinding of participants and personnel (performance bias)	Unclear risk	?
Blinding of outcome assessment (detection bias)	Unclear risk	?
Incomplete outcome data (attrition bias)	Unclear risk	?

Sabri 2014 T1D

**Methods**	Before-after, self-control<br>https://www.ncbi.nlm.nih.gov/pmc/articles/PMC4258680/
**Participants**	Iran, 18 children with T1D, 7 M / 11 F, age 3-18 y<br>**Inclusions:** diagnosed type 1 diabetes, non-smokers, non-pregnant and no history of systemic disease<br>**Exclusions:** Those with a history of recent use of vitamin C, non cooperation and diagnosis of new disorders and any type of congenital heart disease, which was found during echocardiography.
**Interventions**	0.25 g/day vitamin C for 1 month
**Outcomes**	LVEF
**Method and timing to measure LVEF**	no description
**Source of data for LVEF**	Table 2
**LVEF at baseline**	65.88%
**Notes**	We contacted Dr. Tavana (second author) by email on 2021-3-11 and 2021-8-1 but we have not received a response.

Risk of bias table

Bias	Authors' judgement	Support for judgement
Random sequence generation (selection bias)	Unclear risk	Participants were controls for themselves
Allocation concealment (selection bias)	Unclear risk	Participants were controls for themselves
Blinding of participants and personnel (performance bias)	Unclear risk	?
Blinding of outcome assessment (detection bias)	Unclear risk	?
Incomplete outcome data (attrition bias)	Unclear risk	?

Sabri 2016

**Methods**	Randomized double-blind placebo-controlled trial, 2014–2015.<br>https://doi.org/10.4103/1735-1995.193510<br>https://www.ncbi.nlm.nih.gov/pmc/articles/PMC5331767<br>https://www.ncbi.nlm.nih.gov/pubmed/28255327
**Participants**	Iran, children with T1D, 40 Participants, 22 M / 18 F, mean age 13 y (SD 4), 20 vit C / 20 placebo.<br>**Inclusion:** 5–18 years, diabetes for at least 5 years, absence of other systemic diseases, no smoking, no pregnancy, normal diet, no vitamin C in previous 3 months.<br>**Exclusion:** development of any new disease during the study, nonintake of vitamin C or placebo during the study for any reason.
**Interventions**	**Vitamin C:** "250 mg of single daily dose of oral Vitamin C" for 6 months (p 2).<br>**Control group:** "a placebo with the same shape and size of Vitamin C tablets and with the same duration " (p 2).
**Outcomes**	LVEF
**Method and timing to measure LVEF**	"echocardiography" (p 2). The method of LVEF measurement by echocardiography was not described.
**Source of data for LVEF**	Table 3: LVEF for both groups for before and after intervention.
**LVEF at baseline**	Vit C 59.73%, Placebo 61,35%
**Notes**	We tried to contact Dr. Ghaffari by email on 2020-3-13 and 2020-3-20 and 2020-11-18 and 2021-3-16 but we did not receive any responses.

Risk of bias table

Bias	Authors' judgement	Support for judgement
Random sequence generation (selection bias)	Low risk	"the patients were distributed into two twenty-member groups through randomized block design" (p 2).<br>Baseline LVEF was 59.73% in the vitamin C group and 61.35% in the placebo group (Table 3). Groups were also balanced for age, sex, blood pressure, height, weight, BMI, duration of diabetes (Table 1), and for fasting blood sugar, random blood sugar, HbA1c, insulin, HDL, LDL, cholesterol, triglycerides (Table 2). However, in Table 3, the baseline level of VCAM (vascular cell adhesion protein) was much higher in the placebo group than in the vitamin C group (189 vs. 111; P<0.001).
Allocation concealment (selection bias)	Low risk	"coded by a third person unaware of the study details while the project researcher was unaware of the drug specifications" (p 2).
Blinding of participants and personnel (performance bias)	Low risk	"The patients were blinded since they were unaware of the type of drug or placebo administered. Moreover, the drug or placebo was similarly supplied by an identical pharmaceutical company and then coded by a third person unaware of the study details while the project researcher was unaware of the drug specifications" (p 2)
Blinding of outcome assessment (detection bias)	Low risk	"coded by a third person unaware of the study details while the project researcher was unaware of the drug specifications" (p 2)
Incomplete outcome data (attrition bias)	Low risk	"During the study period, no patient was excluded from the study, and forty patients participated to the end" (p 3).

Safaei 2017

**Methods**	Randomized trial<br>https://doi.org/10.4103/0971-9784.197834<br>https://www.ncbi.nlm.nih.gov/pmc/articles/PMC5290695<br>https://www.ncbi.nlm.nih.gov/pubmed/28074795
**Participants**	Iran, elective cardiac surgery patients, 45 M / 13 F, mean age: 57 y (SD 9); 29 vit C / 29 control.<br>**Inclusion:** "patients undergoing first-time elective CABG surgery without concomitant procedures".<br>**Exclusion:** "urgent patients, complicated high-risk patients, diabetics, those who needed another heart surgery beside CABG, and if the ischemic time exceeded 120 min".
**Interventions**	**Vitamin C:** "25 mg/kg Vitamin C through pump circulation during surgery" (p 46) corresponds to 1.85 g/day for the mean weight of 73.6 kg of the patients<br>**Control** "No treatment" (p 46).
**Outcomes**	LVEF (text in right-hand column p 47).
**Method and timing to measure LVEF**	Not described
**Source of data for LVEF**	Table 1 reported preoperative and Table 2 reported postoperative LVEF.
**LVEF at baseline**	Vit C 48.4%, Control 49.1%
**Notes**	We tried to contact Dr. Babaei by email on 2020-3-13 and 2020-3-20 and 2021-3-16 but we did not receive any responses.

Risk of bias table

Bias	Authors' judgement	Support for judgement
Random sequence generation (selection bias)	Low risk	"patients ... were randomly assigned to three groups ... using random allocation software" (p 46).
Allocation concealment (selection bias)	Unclear risk	No description
Blinding of participants and personnel (performance bias)	Unclear risk	No description. "No treatment" ... (p 46) indicates that participants and personnel may have known the treatment. That does not directly indicate that knowledge of treatment by the patients would cause bias in such a variable as LVEF, but we classify risk as unclear. As to observation, the statements of blinding are explicit, see below.
Blinding of outcome assessment (detection bias)	Low risk	"All data were collected by an independent research nurse assigned to this research study and were blinded to the groups" (p 46).<br>"All clinical data were collected by an independent end-point assessor team including a cardiologist and a nurse who were assigned to this clinical trial and were blinded to group assignment" (p 47).
Incomplete outcome data (attrition bias)	Low risk	Flow diagram in Fig 1: In the control group, 5/34, and in the vitamin C group, 7/36, dropped out (Figure 1). All withdrawals were because of protocol violations.

Scalzo 2018 healthy

**Methods**	Randomized cross-over trial<br>https://doi.org/10.1186/s13098-018-0306-9<br>https://www.ncbi.nlm.nih.gov/pmc/articles/PMC5813393<br>https://www.ncbi.nlm.nih.gov/pubmed/29456629
**Participants**	USA, Graded exercise to exhaustion, 10 M / 11 F, mean age: 45 y (33-55)<br>**Inclusion:** "sedentary healthy adults ... absence of comorbid conditions" (p 2).<br>**Exclusion:** "Cigarette use within 1 year prior to study" (p 2).
**Interventions**	**Vitamin C:** "a bolus ... over 20 min followed by a drip-infusion ... the total dose of vitamin C administered (bolus + drip) equaled 7.5 g" (p 3).<br>**Control** "An equal volume of saline was infused during the saline visit to control for plasma volume" (p 3).
**Outcomes**	LVEF<br>Secondary outcomes:<br>Left ventricular circumferential strain (measure of systolic function)<br>Left ventricular longitudinal strain (measure of systolic function)<br>Lateral E:E' (measure of diastolic function)<br>Septal E:E' (measure of diastolic function)<br>Left ventricular circumferential strain
**Method and timing to measure LVEF**	"echocardiography ... at rest and immediately following completion of the cycle graded exercise tests" (p 5). The method of LVEF measurement by echocardiography was not described.
**Source of data for LVEF**	Table 4 and Fig 3d: change in LVEF caused by exercise.
**LVEF at baseline**	Vit C 67%, Placebo 65%
**Notes**	We were able to contact Dr. Rebecca Scalzo by email on 2020-4-16 and received further information about blinding (below) and numerical results for findings that were published as Figure 3d, see our Supplementary file 2 (email stated: "The P value 0.057 was for the main effect of vitamin C, independent of disease status").

Risk of bias table

Bias	Authors' judgement	Support for judgement
Random sequence generation (selection bias)	Low risk	"participants completed two randomly ordered visits" (p 3). The participants were their own controls and therefore systematic baseline differences are unlikely.
Allocation concealment (selection bias)	Low risk	The participants were their own controls and therefore systematic baseline differences are unlikely. In addition, "with regard to blinding, the participants and research staff were blinded at time of data collection, and statistical analyses were completed with the research staff naïve to condition" (email from Rebecca Scalzo 2020-4-16).
Blinding of participants and personnel (performance bias)	Low risk	"With regard to blinding, the participants and research staff were blinded at time of data collection, and statistical analyses were completed with the research staff naïve to condition" (email from Rebecca Scalzo 2020-4-16).
Blinding of outcome assessment (detection bias)	Low risk	"A cardiologist blinded to the treatment allocation of the participants supervised the acquisition of these echocardiographic data and performed all of the measurements and interpretation" (p 4).
Incomplete outcome data (attrition bias)	Low risk	Paired data.

Scalzo 2018 T2D

**Methods**	Randomized cross-over trial<br>https://doi.org/10.1186/s13098-018-0306-9<br>https://www.ncbi.nlm.nih.gov/pmc/articles/PMC5813393<br>https://www.ncbi.nlm.nih.gov/pubmed/29456629
**Participants**	USA, Graded exercise to exhaustion, 24 M / 7 F, mean age 46 y (33-55)<br>**Inclusion:** "Persons with T2D were included if their diabetes was treated by diet alone, metformin and/or oral anti-diabetic medications and demonstrated adequate glycemic control ... All participants were sedentary ... absence of comorbid conditions" (p 2).<br>**Exclusion:** Cigarette use within 1 y, acute liver disease, distal symmetrical neuropathy, autonomic dysfunction, proteinuria, evidence of heart disease limiting exercise performance, hypertension (p 2).
**Interventions**	**Vitamin C:** "a bolus ... over 20 min followed by a drip-infusion ... the total dose of vitamin C administered (bolus + drip) equaled 7.5 g" (p 3).<br>**Control** "An equal volume of saline was infused during the saline visit to control for plasma volume" (p 3).
**Outcomes**	LVEF<br>Secondary outcomes:<br>Left ventricular circumferential strain (measure of systolic function)<br>Left ventricular longitudinal strain (measure of systolic function)<br>Lateral E:E' (measure of diastolic function)<br>Septal E:E' (measure of diastolic function)<br>Left ventricular circumferential strain
**Method and timing to measure LVEF**	"echocardiography ... at rest and immediately following completion of the cycle graded exercise tests" (p 5).<br>The method of LVEF measurement by echocardiography was not described.
**Source of data for LVEF**	Table 4 and Fig 3d: change in LVEF caused by exercise.
**LVEF at baseline**	Vit C 63%, Placebo 65%
**Notes**	We were able to contact Dr. Rebecca Scalzo by email on 2020-4-16 and received further information about blinding (below) and numerical results for findings that were published as Figure 3d, see our Supplementary file 2 (email stated: "The P value 0.057 was for the main effect of vitamin C, independent of disease status").

Risk of bias table

Bias	Authors' judgement	Support for judgement
Random sequence generation (selection bias)	Low risk	"participants completed two randomly ordered visits" (p 3).<br>The participants were their own controls and therefore systematic baseline differences are unlikely.
Allocation concealment (selection bias)	Low risk	The participants were their own controls and therefore systematic baseline differences are unlikely.<br>In addition, "with regard to blinding, the participants and research staff were blinded at time of data collection, and statistical analyses were completed with the research staff naïve to condition" (email from Rebecca Scalzo 2020-4-16).
Blinding of participants and personnel (performance bias)	Low risk	"With regard to blinding, the participants and research staff were blinded at time of data collection, and statistical analyses were completed with the research staff naïve to condition" (email from Rebecca Scalzo 2020-4-16).
Blinding of outcome assessment (detection bias)	Low risk	"A cardiologist blinded to the treatment allocation of the participants supervised the acquisition of these echocardiographic data and performed all of the measurements and interpretation" (p 4).
Incomplete outcome data (attrition bias)	Low risk	Paired data.

Table S2

Table S2: Extraction of results of the included trials

Trial [ref]	Source of data	Baseline						After intervention			
		Control			Vit C			Control		Vit C	
		N	Mean	SD [SE]	N	Mean	SD [SE]	Mean	SD [SE]	Mean	SD [SE]
Basili (2010)[T1,T2]	p 225	28	53.7	3.9	28	52.3	4.3	54.1	4.7	58.3	2.9
Emadi (2019)[T3]	Tables 1,4	25	56.50	6.12	25	56.29	6.29	51.80	6.59	56.80	4.05
Fernhall (2010)[T14-T16]	Abstract	35	61	8	34	58	9	55	9	53	10
Gao (2012)[T4]	Table 2	8	60.38	[1.46]	8	58.63	[1.84]	57.13	[1.63]	57.13	[1.55]
Glavas (2009)[T12]	Table 1	8	66.6	6.1	8	65.8	5.7	62.6	4.6	61.4	3.9
Guan (1999)[T5]	p 925	11	49	[4]	10	51	[4]	51	[4]	55	[6]
Ho (2007)[T6]	p 101: Gr. I	19	34	13				37	13	47	13
	p 101: Gr. II	18	36	9				39	10	47	11
Jensen (1997)[T7]	Fig 4: 6 months				9	55.9				+ 7.0	
Oktar (2001)[T8]	Separate calculation Table III	12	64.00	6.55	12	59.00	4.47	65.83	5.94	67.00	6.41
Sabri (2014) [T13]	Gr. II+IV, day7 Table 2				19	64.44	6.37			64.50	5.68
Sabri (2014) [T13] **T1D**	Table 2				18	65.88	6.69			65.35	5.60
Sabri (2016)[T9] T1D	Table 3	20	61.35	3.40	20	59.73	2.48	59.90	3.15	63.80	5.39
Safaei (2017)[T10]	Tables 1,2	29	49.1	[1.2]	29	48.4	[1.3]	41.7	[1.5]	49.7	[1.0]
Scalzo (2018)[T11]	Table 4 and email	21	65	[2]	21	67	[2]	+16.98		+18.99	
Scalzo (2018)[T11] **T2D**	Table 4 and email	31	65	[1]	31	63	[1]	+18.65		+22.99	

Figure S1: Comparison of vitamin C and control arms

	**Vitamin C**	**Control**
Time 1	LVEF(VitC,1)	LVEF(Control,1)
↓	↓	↓
Time 2	LVEF(VitC,2)	LVEF(Control,2)

Change over time within the arm:

change-LVEF(vitC) = LVEF(VitC,2) – LVEF(VitC,1)

change-LVEF(Control) = LVEF(Control,2) – LVEF(Control,1)

Difference between the trial arms:

Δ -LVEF = change-LVEF(vitC) – change-LVEF(Control)

When SDs for the four groups are available, the SD for the Δ-LVEF can be calculated from the four SD[LVEF(i,j)] values as follows:

$Var = SD^2$

Var(Pooled) = Var(VitC,1) + Var(VitC,2) + Var(Control,1) + Var(Control,2)

$SD(Pooled) = \sqrt{Var(Pooled)}$

Calculation of the P-value for the interaction between vitamin C and time in the LVEF trials

The total sum of squares for a trial on vitamin C for LVEF is SS_{Total}

(1) $SS_{Total} = \Sigma(x_{ijk} - \dot{x})^2$

$= \Sigma[(x_{ijk} - \ddot{x}_{4jk}) - (\dot{x} - \ddot{x}_{4jk})]^2$

$= \Sigma[\, (x_{ijk} - \ddot{x}_{4jk})^2 - 2\cdot(x_{ijk} - \ddot{x}_{4jk})\cdot(\dot{x} - \ddot{x}_{4jk}) + (\dot{x} - \ddot{x}_{4jk})^2\,]$

$= \Sigma(x_{ijk} - \ddot{x}_{4jk})^2 \quad + 0 \quad + \Sigma(\dot{x} - \ddot{x}_{4jk})^2$

Here:

x_{ijk} indicates the individual observations

i indicates the person. The number of participants in all four groups is "n" and it is the same in all included LVEF trials, but there is a different "n" for each trial.

j indicates the group with value 0 or 1 (control or vitamin C).

k indicates time with value 0 or 1 (pre-value or post-value).

$\ddot{x}_{4jk}$ indicates the mean outcome within the 4 groups indexed by jk. These are reported in the LVEF trial reports.

$\dot{x}$ indicates the mean over all the 4 groups: $\dot{x} = (\ddot{x}_{00} + \ddot{x}_{01} + \ddot{x}_{10} + \ddot{x}_{11})/4$.

Since $\ddot{x}_{4jk}$ are the means for x_{ijk} within the 4 groups, by definition the term $\Sigma(x_{ijk} - \ddot{x}_{4jk})$ sums up to value 0 and therefore the middle term in lowest line of formula (1) vanishes. Thus,

(2) $SS_{Total} = \Sigma(x_{ijk} - \dot{x})^2 = \Sigma(x_{ijk} - \ddot{x}_{4jk})^2 + \Sigma(\dot{x} - \ddot{x}_{4jk})^2 = SS_{Within} + SS_{Between}$

The within-group sum of squares SS_W can be calculated from the published SD-values, and from the SE-values, since $SD = SE\cdot\sqrt{n}$.

(3) $SS_{Within} = \Sigma(x_{ijk} - \ddot{x}_{4jk})^2 = (n-1)\cdot Var = (n-1)\cdot SD^2$

The $SS_{Between}$ for the 4 groups can be further decomposed to SS for the model without interaction (3 df) and the interaction (1 df)

(4) $SS_{Between} = \Sigma(\dot{x} - \ddot{x}_{4jk})^2 = \Sigma[(\dot{x} - \ddot{x}_{3jk}) + (\ddot{x}_{3jk} - \ddot{x}_{4jk})]^2$

$= \Sigma[(\dot{x} - \ddot{x}_{3jk})^2 + 2\cdot(\dot{x} - \ddot{x}_{3jk})\cdot(\ddot{x}_{3jk} - \ddot{x}_{4jk}) + (\ddot{x}_{3jk} - \ddot{x}_{4jk})^2\,]$

Here again, the middle term vanishes. Thus,

(5) $SS_{Between} = \Sigma(\dot{x} - \ddot{x}_{3jk})^2 + \Sigma(\ddot{x}_{3jk} - \ddot{x}_{4jk})^2 = SS_{B3df} + SS_{Interaction}$

The $SS_{Interaction}$ can be calculated from the published means within the 4 groups of observations ($\ddot{x}_{4jk}$).
See the derivation of this formula on the following pages.
The P-value for the interaction can be calculated from the F-distribution.

(6) $SS_{Interaction} = n\cdot(\ddot{x}_{400} - \ddot{x}_{401} - \ddot{x}_{410} + \ddot{x}_{411})^2/4$

(7) $F(1\text{ df}, 4\cdot n-4\text{ df}) = SS_{Interaction} / [SS_{Within}/(4\cdot n-4)]$

The interaction P-value is calculated in spreadsheets of Supplement 2.

All the published LVEF trials published mean values for the 4 groups by time and vit C as follows:

Table 1: Published mean values for the 4 groups (4 degrees of freedom)

$\ddot{x}_{400}$	$\ddot{x}_{401}$
$\ddot{x}_{410}$	$\ddot{x}_{411}$

This model has 4 degrees of freedom, based on parameters (means) $\ddot{x}_{400}$, $\ddot{x}_{401}$, $\ddot{x}_{410}$, $\ddot{x}_{411}$.

The following calculations are done using **a, b, c, d** since it is easier to visualize them than $\ddot{x}_{4jk}$.

The sum of squares for the 2-variable linear model with group and time as explanatory variables can be calculated from the published mean values with simple arithmetic as follows on this and next pages.

Table 2: Reported data with 4 degrees of freedom

			Marginal means:
Observations:	a	b	(a + b)/2
	c	d	(c + d)/2
Marginal means:	(a + c)/2	(b + d)/2	

Difference in the marginal means:

DCol = (b + d)/2 – (a + c)/2 for the columns
DRow = (c + d)/2 – (a + b)/2 for the rows

Table 3: The 2-variable linear model with 3 degrees of freedom, based on parameters $\dot{x}$, y, z.

			Marginal means:
3 parameter model:	$\dot{x} - y - z$	$\dot{x} + y - z$	$(2\cdot\dot{x} - 2\cdot z)/2$
	$\dot{x} - y + z$	$\dot{x} + y + z$	$(2\cdot\dot{x} + 2\cdot z)/2$
Marginal means:	$(2\cdot\dot{x} - 2\cdot y)/2$	$(2\cdot\dot{x} + 2\cdot y)/2$	

Difference in the marginal means:

DCol $= (2\cdot\dot{x} + 2\cdot y)/2 - (2\cdot\dot{x} - 2\cdot y)/2 = \dot{x} + y - (\dot{x} - y) = 2\cdot y$ for the columns
DRow $= (2\cdot\dot{x} + 2\cdot z)/2 - (2\cdot\dot{x} - 2\cdot z)/2 = \dot{x} + z - (\dot{x} - z) = 2\cdot z$ for the rows

Comparison of the Tables 2 and 3 gives

$2\cdot y = (b + d)/2 - (a + c)/2 = (b + d - a - c)/2$
$y = (b + d - a - c)/4$

$2\cdot z = (c + d)/2 - (a + b)/2 = (c + d - a - b)/2$
$z = (c + d - a - b)/4$

The y and z calculated on the previous page can be put into the 2-variable linear model as follows:

Table 4: The model with time and treatment without interaction (3 degrees of freedom)

$\ddot{x}_{300} = \dot{x} - y - z$ (00)	$\ddot{x}_{301} = \dot{x} + y - z$ (01)
$\ddot{x}_{310} = \dot{x} - y + z$ (10)	$\ddot{x}_{311} = \dot{x} + y + z$ (11)

$\dot{x} = (\ a + b + c + d)/4$
$y = (-a + b - c + d)/4$
$z = (-a - b + c + d)/4$

Thus, the cells of the table become

(00): $\ddot{x}_{300} = \dot{x} - y - z = (a + b + c + d)/4 - (-a + b - c + d)/4 - (-a - b + c + d)/4$
$= (3 \cdot a + b + c - d)/4$

(01): $\ddot{x}_{301} = \dot{x} + y - z = (a + b + c + d)/4 + (-a + b - c + d)/4 - (-a - b + c + d)/4$
$= (3 \cdot b + a + d - c)/4$

(10): $\ddot{x}_{310} = \dot{x} - y + z = (a + b + c + d)/4 - (-a + b - c + d)/4 + (-a - b + c + d)/4$
$= (3 \cdot c + a + d - b)/4$

(11): $\ddot{x}_{311} = \dot{x} + y + z = (a + b + c + d)/4 + (-a + b - c + d)/4 + (-a - b + c + d)/4$
$= (3 \cdot d + b + c - a)/4$

The SS for the interaction test can be calculated with the following formula, compare (5):

$SS_{Interaction} = \Sigma(\ddot{x}_{4jk} - \ddot{x}_{3jk})^2 = \Sigma\ (\Delta_{jk})^2$

(00): $\Delta_{00} = \ddot{x}_{400} - \ddot{x}_{300} = a - (3 \cdot a + b + c - d)/4 \qquad = \ (a - b - c + d)/4$

(01): $\Delta_{01} = \ddot{x}_{401} - \ddot{x}_{301} = b - (3 \cdot b + a + d - c)/4 = (b - a - d + c)/4 \quad = -(a - b - c + d)/4 = -1 \cdot \Delta_{00}$

(10): $\Delta_{10} = \ddot{x}_{410} - \ddot{x}_{310} = c - (3 \cdot c + a + d - b)/4 = (c - a - d + b)/4 \quad = -(a - b - c + d)/4 = -1 \cdot \Delta_{00}$

(11): $\Delta_{11} = \ddot{x}_{411} - \ddot{x}_{311} = d - (3 \cdot d + b + c - a)/4 = (d - b - c + a)/4 \quad = \ (a - b - c + d)/4 = +1 \cdot \Delta_{00}$

The difference in the SS between the 3 df model and the 4 df model is as follows:

$$\mathbf{SS_{Interaction} = \Sigma(\Delta_{jk})^2 = 4 \cdot n \cdot (\Delta_{00})^2 = 4 \cdot n \cdot (a - b - c + d)^2/(4 \cdot 4) = n \cdot (-\ddot{x}_{400} + \ddot{x}_{401} + \ddot{x}_{410} - \ddot{x}_{411})^2/4}$$

The $SS_{Interaction}$ has 1 degree of freedom (4 df – 3 df).

Printouts of statistical calculations

Meta-analysis of 12 trials by subgroups (cardiac, non-cardiac)

```
> EF_MA <- metagen(RelDif, RelSE, Trial,data=EF, comb.random=F, byvar=
    Context)
> EF_MA
                                          95%-CI %W(fixed)     Context
Basili 2010         10.5660 [  4.7821; 16.3499]      18.2     Cardiac
Emadi 2019           9.2380 [  0.9826; 17.4934]       8.9     Cardiac
Fernhall 2010        1.6810 [ -8.3932; 11.7552]       6.0 Non-cardiac
Gao 2012             2.9410 [ -7.8956; 13.7776]       5.2 Non-cardiac
Glavas 2009         -0.6040 [-11.4818; 10.2738]       5.1 Non-cardiac
Guan 1999            4.0000 [-31.1304; 39.1304]       0.5     Cardiac
Ho 2007             25.8110 [  9.7569; 41.8651]       2.4     Cardiac
Oktar 2001          10.0330 [ -1.0839; 21.1499]       4.9     Cardiac
Sabri 2016           9.1180 [  3.4635; 14.7725]      19.1 Non-cardiac
Safaei 2017         17.8460 [  7.4072; 28.2848]       5.6     Cardiac
Scalzo 2018 Healthy  3.0450 [ -3.9638; 10.0538]      12.4 Non-cardiac
Scalzo 2018 T2D      6.7810 [ -0.4473; 14.0093]      11.7 Non-cardiac

Number of studies combined: k = 12

                                     95%-CI    z  p-value
Fixed effect model 8.0016 [5.5331; 10.4700] 6.35 < 0.0001

Quantifying heterogeneity:
 tau^2 = 9.2052 [0.0000; 90.0237]; tau = 3.0340 [0.0000; 9.4881]
 I^2 = 31.7% [0.0%; 65.5%]; H = 1.21 [1.00; 1.70]

Test of heterogeneity:
     Q d.f. p-value
 16.10   11  0.1375

Results for subgroups (fixed effect model):
                      k                      95%-CI    Q  I^2  tau^2    tau
Context = Cardiac     6 12.0217 [8.1446; 15.8988] 5.03 0.7% 0.1829 0.4277
Context = Non-cardiac 6  5.2612 [2.0601;  8.4622] 4.12 0.0%      0      0


Test for subgroup differences (fixed effect model):
                  Q d.f. p-value
Between groups 6.95    1  0.0084
Within groups  9.15   10  0.5177

Details on meta-analytical method:
- Inverse variance method
- DerSimonian-Laird estimator for tau^2
- Jackson method for confidence interval of tau^2 and tau
```

Meta-analysis of cardiac trials

```
> Cardi_MA <- metagen(RelDif, RelSE, Trial,data=Cardi, comb.random=F)
> Cardi_MA
                                   95%-CI %W(fixed)
Basili 2010 10.5660 [  4.7821; 16.3499]      44.9
Emadi 2019   9.2380 [  0.9826; 17.4934]      22.1
Guan 1999    4.0000 [-31.1304; 39.1304]       1.2
Ho 2007     25.8110 [  9.7569; 41.8651]       5.8
Oktar 2001  10.0330 [ -1.0839; 21.1499]      12.2
Safaei 2017 17.8460 [  7.4072; 28.2848]      13.8

Number of studies combined: k = 6

                                        95%-CI    z  p-value
Fixed effect model 12.0217 [8.1446; 15.8988] 6.08 < 0.0001

Quantifying heterogeneity:
 tau^2 = 0.1829 [0.0000; >100.0000]; tau = 0.4277 [0.0000; >10.0000]
 I^2 = 0.7% [0.0%; 74.8%]; H = 1.00 [1.00; 1.99]

Test of heterogeneity:
    Q d.f. p-value
 5.03    5  0.4118

> 2*(pnorm(-6.08, 0, 1))
[1] 1.2e-09
```

Meta-analysis of 4 methodologically satisfactory cardiac trials

```
> Cardi4_MA <- metagen(RelDif, RelSE, Trial,data=Cardi4, comb.random=F)
> Cardi4_MA
                                   95%-CI %W(fixed)
Basili 2010 10.5660 [  4.7821; 16.3499]      60.7
Emadi 2019   9.2380 [  0.9826; 17.4934]      29.8
Guan 1999    4.0000 [-31.1304; 39.1304]       1.6
Ho 2007     25.8110 [  9.7569; 41.8651]       7.9

Number of studies combined: k = 4

                                        95%-CI    z  p-value
Fixed effect model 11.2633 [6.7575; 15.7690] 4.90 < 0.0001

Quantifying heterogeneity:
 tau^2 = 5.9663 [0.0000; >100.0000]; tau = 2.4426 [0.0000; >10.0000]
 I^2 = 16.8% [0.0%; 87.3%]; H = 1.10 [1.00; 2.80]

Test of heterogeneity:
    Q d.f. p-value
 3.61    3  0.3073

> 2*(pnorm(-4.90, 0, 1))
[1] 9.5-07
```

Meta-analysis of non-cardiac trials

```
> NonCardi_MA <- metagen(RelDif, RelSE, Trial,data=NonCardi, comb.random=F)
> NonCardi_MA
                                                  95%-CI %W(fixed)
Fernhall 2010          1.6810 [ -8.3932; 11.7552]      10.1
Gao 2012               2.9410 [ -7.8956; 13.7776]       8.7
Glavas 2009           -0.6040 [-11.4818; 10.2738]       8.7
Sabri 2016             9.1180 [  3.4635; 14.7725]      32.0
Scalzo 2018 Healthy  3.0450 [ -3.9638; 10.0538]      20.9
Scalzo 2018 T2D        6.7810 [ -0.4473; 14.0093]      19.6

Number of studies combined: k = 6

                                    95%-CI    z p-value
Fixed effect model 5.2612 [2.0601; 8.4622] 3.22  0.0013

Quantifying heterogeneity:
 tau^2 = 0 [0.0000; 62.8584]; tau = 0 [0.0000; 7.9283]
 I^2 = 0.0% [0.0%; 74.6%]; H = 1.00 [1.00; 1.99]

Test of heterogeneity:
    Q d.f. p-value
 4.12    5  0.5324
```

Meta-regression comparison of po and iv (route)

```
> MetaReg_route <- metareg(EF_MA,route)
> MetaReg_route

Mixed-Effects Model (k = 12; tau^2 estimator: DL)

tau^2 (estimated amount of residual heterogeneity):     12.6587 (SE =
    15.4700)
tau (square root of estimated tau^2 value):             3.5579
I^2 (residual heterogeneity / unaccounted variability): 37.48%
H^2 (unaccounted variability / sampling variability):   1.60
R^2 (amount of heterogeneity accounted for):            0.00%

Test for Residual Heterogeneity:
QE(df = 10) = 15.9956, p-val = 0.0998

Test of Moderators (coefficient 2):
QM(df = 1) = 0.0884, p-val = 0.7662

Model Results:

         estimate      se     zval    pval    ci.lb    ci.ub
intrcpt    8.3307  2.0857   3.9942  <.0001   4.2428  12.4185  ***
routepo   -1.0940  3.6799  -0.2973  0.7662  -8.3064   6.1183
```

Meta-regression by dose

```
> MetaReg_Dose <- metareg(EF_MA,dose)
> MetaReg_Dose

Mixed-Effects Model (k = 12; tau^2 estimator: DL)

tau^2 (estimated amount of residual heterogeneity):     11.8408 (SE =
    15.1493)
tau (square root of estimated tau^2 value):             3.4410
I^2 (residual heterogeneity / unaccounted variability): 35.93%
H^2 (unaccounted variability / sampling variability):   1.56
R^2 (amount of heterogeneity accounted for):            0.00%

Test for Residual Heterogeneity:
QE(df = 10) = 15.6078, p-val = 0.1114

Test of Moderators (coefficient 2):
QM(df = 1) = 0.1164, p-val = 0.7330

Model Results:

         estimate      se     zval    pval    ci.lb    ci.ub
intrcpt    8.6581  2.6210   3.3033  0.0010   3.5210  13.7953  ***
dose      -0.1755  0.5145  -0.3412  0.7330  -1.1840   0.8329
```

Meta-regression of 12 trials by baseline LVEF level in the vitamin C group

```
> MetaReg_EF <- metareg(EF_MA,baseEF)
> MetaReg_EF

Mixed-Effects Model (k = 12; tau^2 estimator: DL)

tau^2 (estimated amount of residual heterogeneity):     0 (SE = 8.9580)
tau (square root of estimated tau^2 value):             0
I^2 (residual heterogeneity / unaccounted variability): 0.00%
H^2 (unaccounted variability / sampling variability):   1.00
R^2 (amount of heterogeneity accounted for):            100.00%

Test for Residual Heterogeneity:
QE(df = 10) = 4.7576, p-val = 0.9068

Test of Moderators (coefficient 2):
QM(df = 1) = 11.3403, p-val = 0.0008

Model Results:

         estimate       se     zval    pval    ci.lb    ci.ub
intrcpt   46.6476  11.5450   4.0405  <.0001  24.0199  69.2753  ***
baseEF    -0.6640   0.1972  -3.3675  0.0008  -1.0504  -0.2775  ***



> #Calculation of regression line crossing the null level
>
> (46.6476/0.6640)
[1] 70.25241
```

Meta-regression excluding the Ho trial

```
> NOHO_MA <- metagen(RelDif, RelSE, Trial,data=NoHo, comb.random=F)
> NOHO_MA
                                         95%-CI %W(fixed)
Basili 2010         10.5660 [  4.7821; 16.3499]      18.7
Emadi 2019           9.2380 [  0.9826; 17.4934]       9.2
Fernhall 2010        1.6810 [ -8.3932; 11.7552]       6.1
Gao 2012             2.9410 [ -7.8956; 13.7776]       5.3
Glavas 2009         -0.6040 [-11.4818; 10.2738]       5.3
Guan 1999            4.0000 [-31.1304; 39.1304]       0.5
Oktar 2001          10.0330 [ -1.0839; 21.1499]       5.0
Sabri 2016           9.1180 [  3.4635; 14.7725]      19.5
Safaei 2017         17.8460 [  7.4072; 28.2848]       5.7
Scalzo 2018 Healthy  3.0450 [ -3.9638; 10.0538]      12.7
Scalzo 2018 T2D      6.7810 [ -0.4473; 14.0093]      11.9

Number of studies combined: k = 11

                                     95%-CI    z  p-value
Fixed effect model 7.5703 [5.0722; 10.0685] 5.94 < 0.0001

Quantifying heterogeneity:
 tau^2 = 2.3371 [0.0000; 50.3749]; tau = 1.5288 [0.0000; 7.0975]
 I^2 = 11.2% [0.0%; 51.3%]; H = 1.06 [1.00; 1.43]

Test of heterogeneity:
     Q d.f. p-value
 11.26   10  0.3379

Details on meta-analytical method:
- Inverse variance method
- DerSimonian-Laird estimator for tau^2
- Jackson method for confidence interval of tau^2 and tau
> NOHOReg <- metareg(NOHO_MA,MeanEF)
> NOHOReg

Mixed-Effects Model (k = 11; tau^2 estimator: DL)

tau^2 (estimated amount of residual heterogeneity):     0 (SE = 9.1069)
tau (square root of estimated tau^2 value):             0
I^2 (residual heterogeneity / unaccounted variability): 0.00%
H^2 (unaccounted variability / sampling variability):   1.00
R^2 (amount of heterogeneity accounted for):            100.00%

Test for Residual Heterogeneity:
QE(df = 9) = 4.7114, p-val = 0.8587

Test of Moderators (coefficient 2):
QM(df = 1) = 6.5446, p-val = 0.0105

Model Results:

         estimate      se     zval    pval    ci.lb    ci.ub
intrcpt   45.2505 14.7840   3.0608  0.0022  16.2744  74.2266  **
MeanEF    -0.6348  0.2481  -2.5582  0.0105  -1.1211  -0.1484   *
```
